# Caloric Restriction Rejuvenates Skeletal Muscle Growth in Heart Failure With Preserved Ejection Fraction

**DOI:** 10.1016/j.jacbts.2023.09.014

**Published:** 2023-12-06

**Authors:** Ever Espino-Gonzalez, Peter G. Tickle, Raffaele Altara, Harrison Gallagher, Chew W. Cheng, Viktor Engman, Nathanael Wood, Gustavo Jose Justo da Silva, Mattia Scalabrin, Xinyue Yu, Ziyi Zhong, Michael A. Colman, Nadira Y. Yuldasheva, George W. Booz, Volker Adams, Marcelo G. Pereira, Alessandro Cataliotti, Lee D. Roberts, Stuart Egginton, T. Scott Bowen

**Affiliations:** a School of Biomedical Sciences, Faculty of Biological Sciences, University of Leeds, Leeds, United Kingdom; b Department of Anatomy & Embryology, Faculty of Health, Medicine and Life Sciences, Maastricht University, Maastricht, the Netherlands; c Department of Pathology, School of Medicine, University of Mississippi Medical Center, Jackson, Mississippi, USA; d Leeds Institute of Cardiovascular and Metabolic Medicine, Faculty of Medicine, University of Leeds, Leeds, United Kingdom; e Institute for Experimental Medical Research, Oslo University Hospital and University of Oslo, Oslo, Norway; f Department of Pharmacology and Toxicology, School of Medicine, University of Mississippi Medical Center, Jackson, Mississippi, USA; g Heart Center Dresden, TU-Dresden, Dresden, Germany

**Keywords:** diet, exercise training, HFpEF, mitochondria, skeletal muscle

## Abstract

Heart failure with preserved ejection fraction (HFpEF) is a major clinical problem, with limited treatments. HFpEF is characterized by a distinct, but poorly understood, skeletal muscle pathology, which could offer an alternative therapeutic target. In a rat model, we identified impaired myonuclear accretion as a mechanism for low myofiber growth in HFpEF following resistance exercise. Acute caloric restriction rescued skeletal muscle pathology in HFpEF, whereas cardiac therapies had no effect. Mechanisms regulating myonuclear accretion were dysregulated in patients with HFpEF. Overall, these findings may have widespread implications in HFpEF, indicating combined dietary with exercise interventions as a beneficial approach to overcome skeletal muscle pathology.

## Highlights

• HFpEF is characterized by a distinct, but poorly understood, skeletal muscle pathology, which is emerging as an alternative therapeutic target.
• Using a rat model, we first demonstrated that clinical drugs improving cardiac function did not rescue skeletal muscle pathology. Surprisingly, using a local exercise intervention, we next identified a previously unknown mechanistic deficit in HFpEF that showed failure to increase muscle growth.
• We then discovered that acute dietary caloric restriction restored muscle growth in HFpEF in combination with exercise intervention, which mechanistically could be explained via increasing myonuclear accretion and restoring myogenic homeostasis.
• Given we found similar mechanisms dysregulated in muscle tissue from patients with HFpEF, our findings indicate combining dietary restriction with exercise could be an optimal approach to rescue skeletal muscle pathology in HFpEF that should be further investigated.

## Visual Abstract

**Figure:**
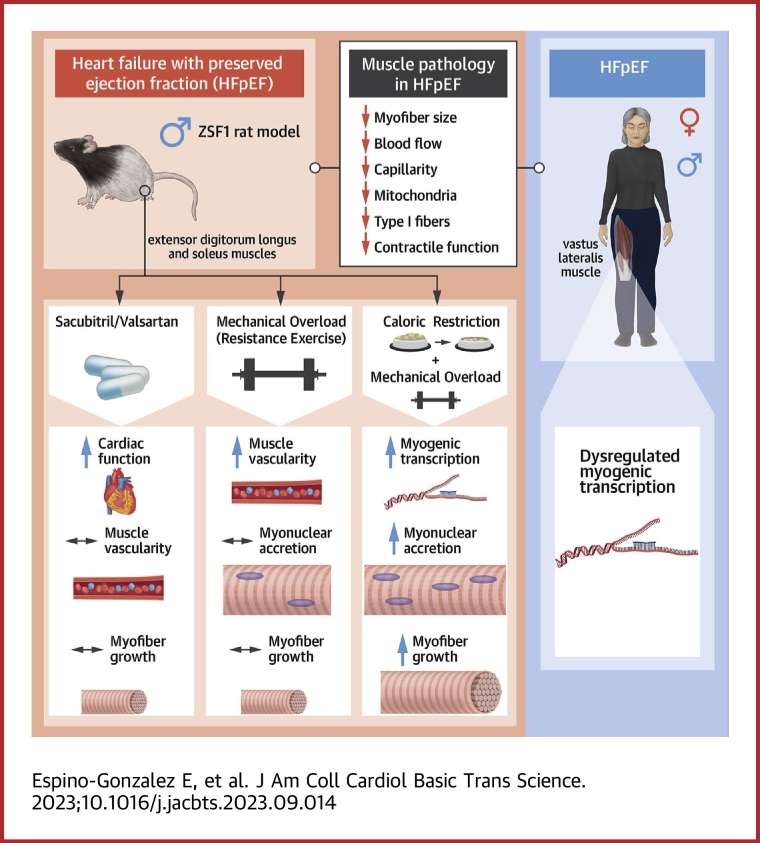

Heart failure is an incurable disease for millions of people worldwide, one-half of whom die within 5 years of diagnosis, and rates continue to rise.^1^ Heart failure characterized by preserved ejection fraction (HFpEF) is rapidly becoming more frequent than classic heart failure with reduced ejection fraction and represents one of the biggest challenges in modern cardiology.^1^^,^^2^ HFpEF is a complex, systemic syndrome characterized by both cardiac and extracardiac pathologies.^3^^,^^4^ Extracardiac organ dysfunction in HFpEF is central to disease progression, with skeletal muscle considered a key emerging therapeutic target.^3^ Whereas many established cardiocentric pharmacological treatments do not improve quality of life or clinical outcomes in HFpEF,^2^ treating the skeletal muscle pathology could provide an alternative strategy.^3^

Skeletal muscle health has widespread clinical consequences in heart failure, impacting functional,^3^ metabolic,^5^ and mental health^6^ status. Although poor skeletal muscle health in HFpEF is closely associated with symptoms, quality of life, and mortality,^7^ the underlying mechanisms remain poorly defined. Morphological changes include reduced capillarity,^8^ increased fat infiltration,^9^ a fiber-type transition (Type I to Type II),^8^ and mitochondrial abnormalities that increase reliance on fatigue-related anaerobic metabolism.^10^^,^^11^ In particular, reduced skeletal muscle mass (pathological atrophy) is a serious clinical complication in HFpEF, causing frailty and poor prognosis.^12^ Because HFpEF is incurable, preserving skeletal muscle health is critical for patients to maintain an acceptable quality of life.^3^ One way to achieve this is by increasing muscle mass or myofiber growth (physiological hypertrophy), which is determined by 2 nonexclusive mechanisms: 1) elevated protein synthesis via Akt-mTOR signaling; and/or 2) addition of new myonuclei via muscle stem cell (MuSC) recruitment.^13^ It currently remains unclear why patients with HFpEF have decreased muscle mass, but studies in both patients and animal models have focused on mechanisms related to myofiber atrophy^14^, ^15^, ^16^, ^17^, ^18^ rather than myofiber growth.

At present, there are few treatments for the skeletal muscle pathology in HFpEF. Whereas many pharmacological treatments targeting the cardiovascular system have been clinically neutral,^2^ it remains unclear whether they provide secondary benefits to skeletal muscle health. Apart from exercise training,^19^ dietary interventions such as caloric restriction (CR) may offer an effective nonpharmacological approach to attenuate muscle pathology in HFpEF and improve quality of life,^5^^,^^20^ CR improves lifespan as well as homeostasis in multiple cells and tissues,^21^ including myofibers^22^ and MuSCs,^23^ however the mechanistic effects of CR on skeletal muscle health in HFpEF remain untested.

Overall, the inability to stimulate skeletal muscle growth in HFpEF has severe clinical consequences.^12^ Here, we applied multiple interventions including pharmacological, exercise, and dietary in a well-established rat model of HFpEF.^24^, ^25^, ^26^, ^27^, ^28^, ^29^, ^30^ Our integrative, multiorgan approach identified decreased overload-induced myofiber growth that was rejuvenated following acute CR as a fundamental feature and potential treatment of skeletal muscle pathology in HFpEF. The mechanism for absent myofiber growth in HFpEF was linked to low myonuclear accretion associated with disturbances in MuSC homeostasis. We further provide evidence that these mechanisms are present in the skeletal muscle of patients with HFpEF. Taken together, our experiments suggest that acute dietary restriction is capable of rejuvenating skeletal muscle health in HFpEF.

## Methods

All protocols and experimental details are outlined in full detail within the Supplemental Appendix. All experiments in animals and humans were ethically approved, and all human participants provided written informed consent. Although past studies indicate that the majority of patients with HFpEF are female,^1^ recent studies now indicate that around 45% are male.^31^ Based upon this, and on evidence that muscle mass and growth can be influenced by female sex hormones (eg, estrogen) that are generally very low in elderly females such as those with HFpEF,^32^ this study selected to use male rats to reduce confounding variables.

### Statistical analysis

Following appropriate checks of normality (Shapiro-Wilks test), differences between control rats and HFpEF were analyzed by unpaired 2-tailed Student *t*-tests; differences among 3 groups were assessed by 1-way analysis of variance followed by Bonferroni post hoc test for multiple pairwise comparisons, and differences for functional overload interventions were assessed by 2-way analysis of variance followed by Bonferroni post hoc test. Analyses were performed in GraphPad Prism (version 9, GraphPad Software). Data are presented as the means ± SD, and a *P* value <0.05 was considered statistically significant.

## Results

### Pharmacological drugs improve cardiac function in HFpEF, but do not rescue skeletal muscle pathology

The obese ZSF1 rat model reflects patient HFpEF-driven cardiac and skeletal muscle pathology.^16^^,^^18^^,^^24^, ^25^, ^26^, ^27^, ^28^, ^29^, ^30^ We^16^^,^^25^^,^^33^ and others^18^ identified skeletal muscle atrophy as a major pathological feature in obese ZSF1 rats that develop HFpEF. After confirming the clinical HFpEF phenotype of this model in comparison to age-matched lean control rats (Supplemental Figure 1), we tested whether improving cardiac function could rescue skeletal muscle pathology in HFpEF.

**Figure 1:**
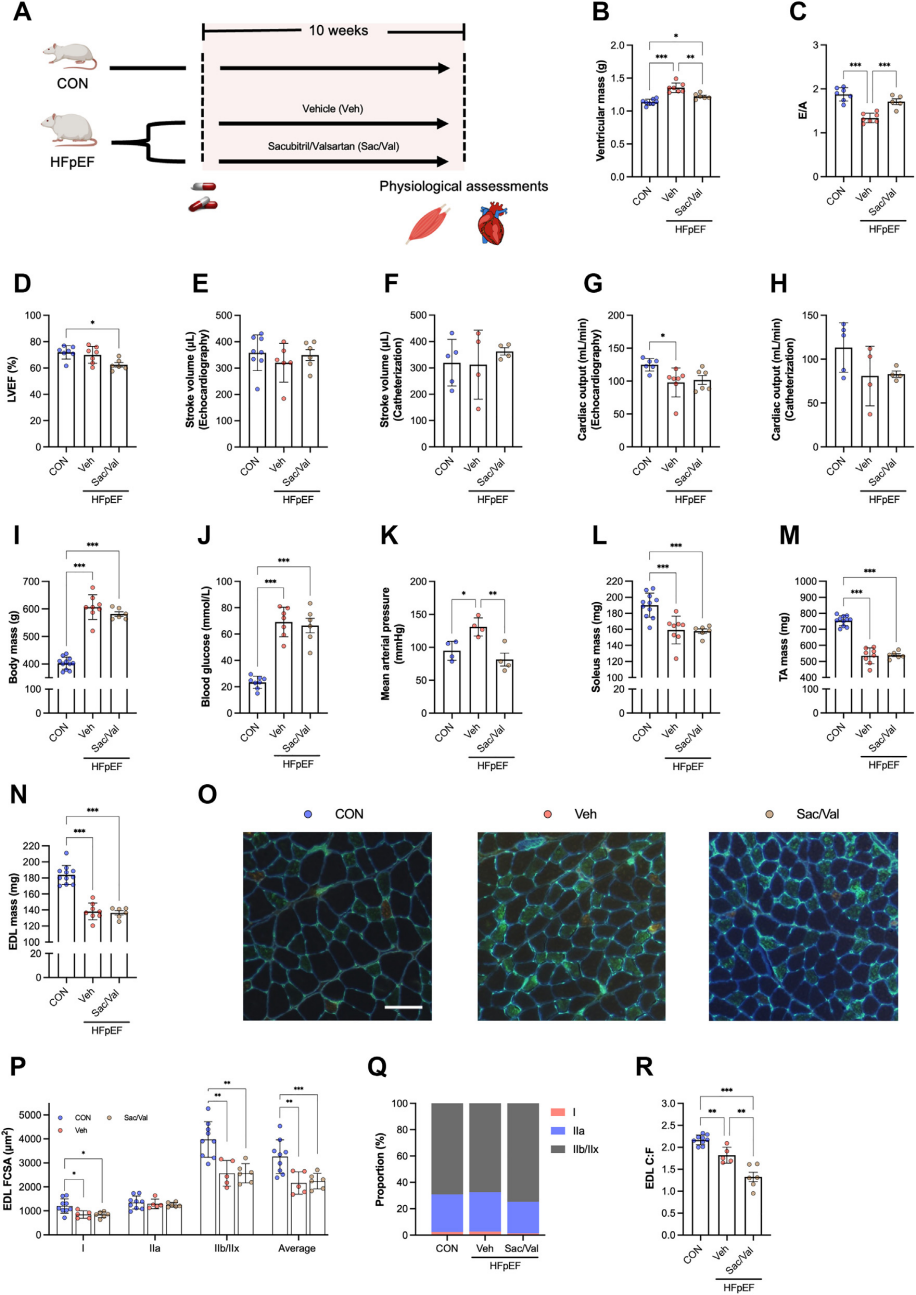
Sac/Val Improves Cardiac Function in HFpEF, But Does Not Attenuate Skeletal Muscle Pathology (A) Schematic of pharmacological treatment in heart failure with preserved ejection fraction (HFpEF) vs control (CON) rats. (B) Ventricular mass after removing atria (CON n = 8, HFpEF+ vehicle [Veh] n = 7, HFpEF+ sacubitril/valsartan [Sac/Val 68 mg/kg body mass/d via gavage] n = 6). (C) Early (E-wave) and late (A-wave) ventricular filling velocities (CON n = 7, HFpEF+Veh n = 7, HFpEF+Sac/Val n = 5), (D) left ventricular ejection fraction (LVEF) (CON n = 7, HFpEF+Veh n = 7, HFpEF+Sac/Val n = 6) (E) Stroke volume (CON n = 8, HFpEF+Veh n = 6, HFpEF+Sac/Val n = 6) and (G) cardiac output (CON n = 6, HFpEF+Veh n = 7, HFpEF+Sac/Val n = 6) assed by noninvasive echocardiography or (F-H) invasive catheterization (CON n = 5, HFpEF+Veh n = 4, HFpEF+Sac/Val n = 4). Metabolic features including (I) body mass (CON n = 11, HFpEF+Veh n = 8, HFpEF+Sac/Val n = 6), (J) blood glucose (CON n = 8, HFpEF+Veh n = 7, HFpEF+Sac/Val n = 6), and (K) mean arterial blood pressure (each group n = 4). (L) Soleus, (M) tibialis anterior (TA), and (N) extensor digitorum longus (EDL) muscles (CON n = 11, HFpEF+Veh n = 8, HFpEF+Sac/Val n = 6) were blotted on paper tissue, and wet mass was recorded. (O) Representative images of extensor digitorum longus (EDL) cryosections stained for Type I (red), Type IIa (green), and Type IIb/IIx (unstained/black) fibers and capillaries (bright green). Histological features of the EDL muscle including (P) total (ie, average) and fiber-type-specific cross-sectional area (FCSA), (Q) numerical proportion, and (R) global capillary-to-fiber ratio (C:F) (CON n = 9, HFpEF+Veh n = 5, HFpEF+Sac/Val n = 6). Between-group differences were assessed by 1-way analysis of variance followed by Bonferroni post hoc test. Data are presented as mean ± SD, and the level of significance was accepted as ∗*P* < 0.05; ∗∗*P* < 0.01; and ∗∗∗*P* < 0.001 for all analyses.

Clinically approved sacubitril/valsartan (Sac/Val) is a neprilysin inhibitor and angiotensin II Type I (AT_1_) receptor blocker that improves cardiac function and remodeling in HFpEF animal models and showed potential benefits in patients.^34^^,^^35^ To date, no study has comprehensively addressed the effects of this drug on myofiber pathology in HFpEF.^29^ Therefore, we evaluated the effects of Sac/Val on the cardiac and skeletal muscle phenotype of obese male ZSF1 HFpEF rats (Figure 1A). Ten weeks of treatment with Sac/Val improved cardiac structure and function in rats with HFpEF, which included reduced ventricular hypertrophy (Figure 1B), improved diastolic function as seen by the normalization of the diastolic mitral inflow E wave to A wave ratio (E/A) using echocardiography (Figure 1C). Whereas systolic function was normal in all groups (Figure 1D), invasive and noninvasive measurements of stroke volume and cardiac output were preserved after drug treatment (Figures 1E to 1H). Sac/Val had no effects on comorbidities such as obesity and hyperglycemia, but reduced hypertension (Figures 1I to 1K).

In contrast to these cardiac effects, Sac/Val treatment did not attenuate skeletal muscle pathology in rats with HFpEF. Gross wet-mass was reduced by ∼15% to 30% across predominant Type I soleus and predominant Type II tibialis anterior and extensor digitorum longus (EDL) muscles in both untreated and treated HFpEF groups compared with control rats (Figures 1L to 1N, Supplemental Figures 2a to 2c). As gross muscle wet-mass does not always reflect myofiber size and provides limited insight into structural changes, we also performed detailed myofiber phenotyping in cryosections from the predominant Type II EDL muscle (Figure 1O). This approach found fiber atrophy in HFpEF compared with control rats independent of Sac/Val treatment (Figure 1P), which was independent of isoform shifts (Figure 1Q) and associated with overt global and local capillary rarefaction (Figure 1R, Supplemental Figures 2d to 2f). Because skeletal muscle remodeling can be influenced by fiber phenotype, we also performed detailed analysis on cryosections from the predominant Type I soleus muscle. This included measuring fiber size, isoform, capillarity, and fibrosis, but we did not find any differences between groups (Supplemental Figures 2g to 2o). These data align well with past studies that demonstrated Type II, rather than Type I, muscles are more sensitive to fiber atrophy and skeletal muscle pathology in heart failure.^36^, ^37^, ^38^ Because heart failure mostly causes pathology in Type II rather than Type I fibers,^36^, ^37^, ^38^, ^39^ we subsequently directed most of our attention to the EDL muscle, given its predominant Type II phenotype, but also performed further analysis on a predominant Type I muscle (soleus) to provide a comprehensive and balanced assessment of muscle fiber types. Overall, these experiments confirm that cardiovascular medications that increase global cardiac function do not increase myofiber growth or rescue skeletal muscle pathology in HFpEF.

### Myofiber growth in HFpEF is impaired following physical loading

**Figure 2:**
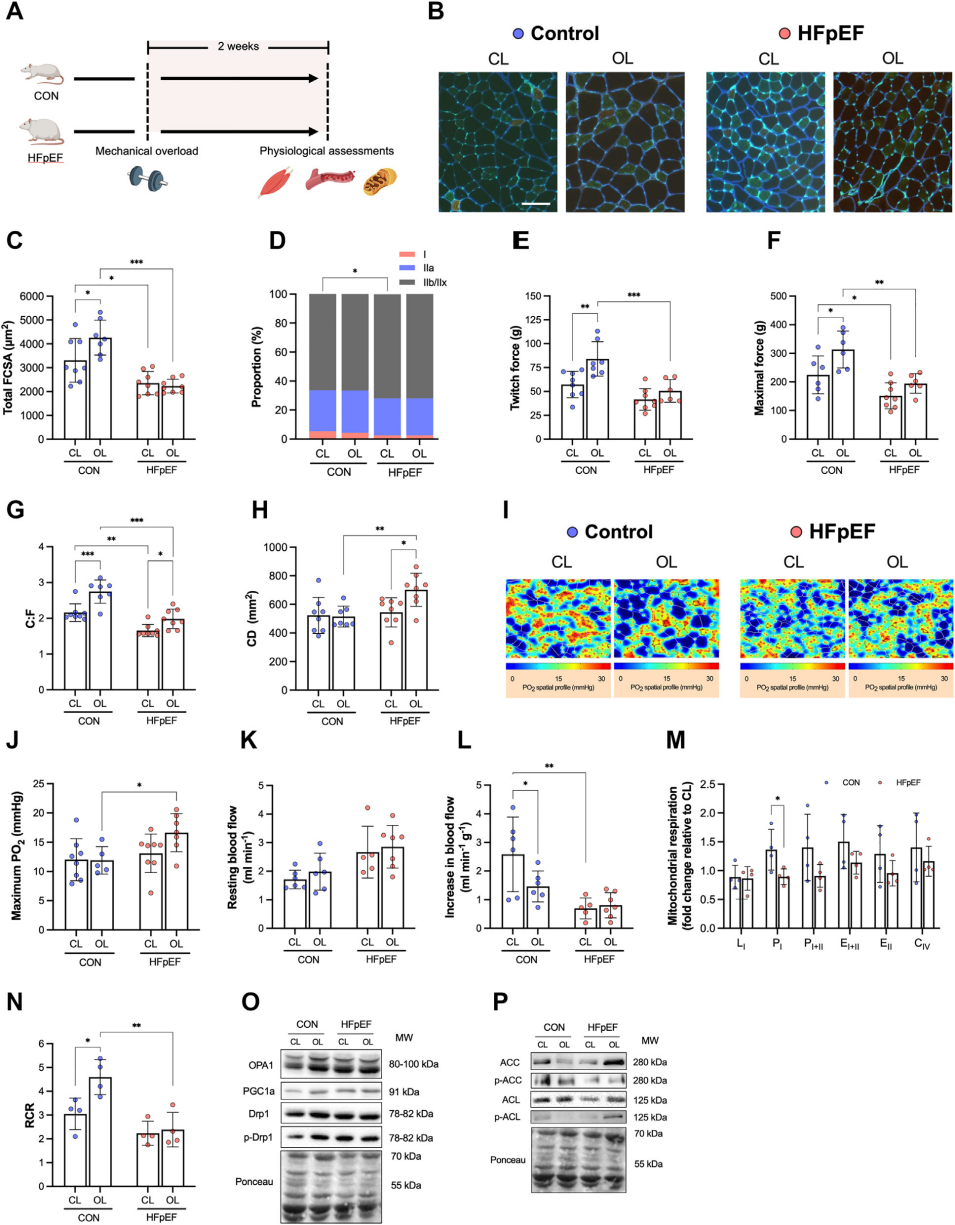
Skeletal Muscle Growth Following Mechanical Overload Is Absent in HFpEF (A) Unilateral synergistic surgical ablation of the tibialis anterior (TA) was performed to induce myofiber hypertrophy in the extensor digitorum longus (EDL) in HFpEF and control (CON) rats. (B) Representative images of nonoverload, contralateral (CL) and overload (OL) EDL muscles stained for Type I (red), Type IIa (green), and Type IIb/IIx (unstained/black) fibers and capillaries (bright green). (C) Total EDL FCSA and (D) fiber type distribution of CL (CON n = 8, HFpEF n = 8) and OL (CON n = 7, HFpEF n = 8). Absolute (E) twitch and (F) maximal forces (CL muscles: CON n = 8 and HFpEF n = 8; OL muscles: CON n = 7 and HFpEF n = 6). (G) Capillary-to-fiber ratio (C:F), (H) capillary density (CD) (CL muscles: CON n = 8 and HFpEF n = 8; OL muscles: CON n = 7 and HFpEF n = 8), and (I and J) muscle oxygen tension at maximal rate of oxygen consumption (CL muscles: CON n = 8 and HFpEF n = 8; OL muscles: CON n = 5 and HFpEF n = 7). In situ femoral artery blood flow at (K) rest and (L) during muscle stimulation (CL muscles: CON n = 6 and HFpEF n = 5; OL muscles: CON n = 6 and HFpEF n = 7). (M) In situ mitochondrial respiratory states of permeabilized EDL fibers presented as fold change relative to baseline CL muscle (each group n = 4). (N) Respiratory control ratio (RCR) (each group n = 4). Protein contents of (O) OPA1, PGC-1α, Drp1, (P) ACC, and ACL (each group n = 4). Differences were assessed by 2-way analysis of variance followed by Bonferroni post hoc test. Data are presented as mean ± SD, and the level of significance was accepted as ∗*P* < 0.05; ∗∗*P* < 0.01; and ∗∗∗*P* < 0.001 for all analyses. C_IV_ = complex IV activity; E_I+II_ = uncoupled respiration in the presence of complex I+II substrates; E_II_ = uncoupled respiration in the presence of complex II substrates; L_I_ = leak respiration with complex I substrates; P_I_ = oxidative phosphorylation with complex I substrates; P_I+II_ = oxidative phosphorylation with complex I+II substrates; other abbreviations as in Figure 1.

Because a cardiocentric approach had no effect on the skeletal muscle pathology in HFpEF, we next used an intervention to mimic resistance exercise and test directly whether a muscle-specific approach could increase myofiber growth. Mechanical overload is a well-accepted model to induce and study the basic mechanisms of load-dependent muscle hypertrophy.^13^ Here, we subjected control and HFpEF male rats to mechanical overload via synergist ablation by surgically removing the tibialis anterior from the right leg, and then evaluated the overload-induced EDL growth after 14 days (Figure 2A). EDL-specific overload provides a more physiologically relevant response compared with optional plantaris overload.^40^ We hypothesized that HFpEF would partially attenuate myofiber growth. Strikingly, detailed analysis of EDL cryosections revealed overload-induced growth increased myofiber size by ∼30% in control rats, an effect that was completely abolished in HFpEF (Figures 2B and 2C). As expected, the effects on myofiber size were preferentially in Type II fibers (Supplemental Figures 3a to 3c). Low myofiber growth in HFpEF was not associated with fiber-isoform shifts (Figure 2D). In line with limited myofiber growth, we confirmed overall muscle function in situ only increased in control rats but not in HFpEF, as demonstrated by EDL absolute twitch and maximal tetanic forces (Figures 2E and 2F). Nevertheless, intrinsic contractile function (force normalized to muscle mass) and relative fatigue were not different among groups (Supplemental Figures 3d to 3f). Overall, these data highlight a novel mechanistic deficit in HFpEF: namely, failure to increase myofiber growth despite adequate physical stimuli. We next explored two key questions: what mechanisms cause, and what treatments could overcome, low myofiber growth in HFpEF.

### Muscle blood flow does not limit load-induced myofiber growth in HFpEF

The skeletal muscle pathology in HFpEF is characterized by peripheral vascular impairments including decreased muscle blood flow and capillarity,^8^^,^^33^ which are important for overload-induced myofiber growth.^41^ We tested whether overload-induced myofiber growth is associated with changes in muscle capillarity in both healthy and HFpEF rats. We evaluated the muscle capillary network in EDL cryosections and then modelled these data to simulate muscle oxygen transport kinetics during resting and maximal metabolic rates.^42^ After overload, global capillary-to-fiber ratio increased in both control and HFpEF groups (Figure 2G). By contrast, global capillary density increased in the HFpEF group only (Figure 2H), which indicates capillary proliferation proceeded at a greater rate than myofiber growth. To provide further insight, we also assessed local fiber type changes in capillarity, which confirmed changes occurred in a Type IIa– and IIx/b–dependent manner (Supplemental Figures 4a to 4f). Furthermore, our in silico simulations identified that overload did not cause significant changes to muscle PO_2_ at rest (Supplemental Figures 4g and 4h) or at maximal metabolic rates in both groups (Figures 2I and 2J).

Muscle blood flow is physiologically dynamic, and this can affect muscle mass and function.^43^^,^^44^ To assess this, we used a unique in situ bilateral limb and femoral artery set up to simultaneously compare both the overload and nonoverload EDL blood flow response in control and HFpEF groups (Supplemental Figures 4i to 4l). Whereas control and HFpEF rats showed no difference in absolute resting leg blood flow after overload (Figure 2K), the functional hyperemia (ie, increase in blood flow relative to muscle mass) in response to stimulated contractions was lower in control rats by 44% but unchanged in HFpEF rats after overload (Figure 2L). Collectively, these data show that muscle capillarity and blood flow respond adequately to the overload stimuli in HFpEF, and indicate other mechanisms are likely responsible for low myofiber growth.

### Impaired mitochondrial adaptation accompanies decreased myofiber growth in HFpEF

HFpEF is characterized by skeletal muscle mitochondrial abnormalities that closely correlate with symptom severity.^10^^,^^11^ Disturbed mitochondrial homeostasis directly impacts myofiber size,^45^ but this relationship has not been explored in HFpEF. Protein synthesis is a highly energetic process, meaning impairments in mitochondrial ATP production could lower cellular energetic state to limit protein synthesis and blunt myofiber hypertrophy.^46^ We determined whether changes in overload-induced hypertrophy are accompanied by alterations in mitochondrial function. In situ mitochondrial high-resolution respirometry experiments in permeabilized EDL fibers showed that, after overload, complex I–dependent respiration increased in control rats relative to the nonoverload leg but was unchanged in HFpEF rats (Figure 2M). We next examined citrate synthase activity in each group as a marker of mitochondrial content but found no effect (Supplemental Figure 5a), identifying mitochondrial function rather than content increased after mechanical overload in control rats, but not HFpEF. This suggestion was further strengthened when we determined the mitochondrial coupling efficiency in the EDL, which increased by 51% in control rats after overload, but no effect was seen in HFpEF (Figure 2N). These data support that mitochondrial functional properties could limit load-induced myofiber growth in HFpEF.

To further explore a molecular mechanism regulating myofiber size related to mitochondrial efficiency and function,^45^ we examined markers of mitochondrial dynamics from the overload vs contralateral EDL of control and HFpEF rats (Figures 2O and 2P, Supplemental Figures 5b to 5l). We found no differences in the expression of pro-fusion and –fission proteins OPA1 and DRP1 between groups, respectively, or no differences in the master regulator of mitochondrial biogenesis PGC1α and its upstream regulator AMPK, as indicated by expression of its target ACC (Figures 2O and 2P, Supplemental Figures 5b to 5i). To explore additional mechanisms, we measured expression of the metabolic enzyme ATP citrate lyase (ACL), which is reduced in sarcopenic mice and links myofiber hypertrophy to mitochondrial function via IGF-AKT signaling.^47^ Whereas basal expression of phosphorylated ACL was lower in HFpEF compared with control rats, which may indicate it could contribute to fiber atrophy (Supplemental Figure 5k), phosphorylation increased after overload in HFpEF rats only (Supplemental Figure 5k to 5l). Taken together, these data suggest that decreased load-induced myofiber growth in HFpEF may be limited, at least in part, by mitochondrial dysfunction.

### Caloric restriction rejuvenates load-induced myofiber growth in HFpEF

Although our data highlight a novel mechanistic deficit in HFpEF related to anabolic resistance, the fundamental mechanisms that impairs myofiber growth and an intervention that rescues myofiber growth in HFpEF are not established. Preliminary evidence from patients indicates that CR could improve skeletal muscle health, especially when combined with exercise training.^5^^,^^20^ For example, CR in young and old age improves skeletal muscle homeostasis,^22^^,^^23^ whereas lifelong CR increases load-induced myofiber growth in aged rats via restored Akt-mTORC1 signaling.^48^ Based on evidence from clinical^5^ and rodent^5^ experiments, we reasoned combining mechanical overload with CR in HFpEF could rejuvenate myofiber growth.

**Figure 3:**
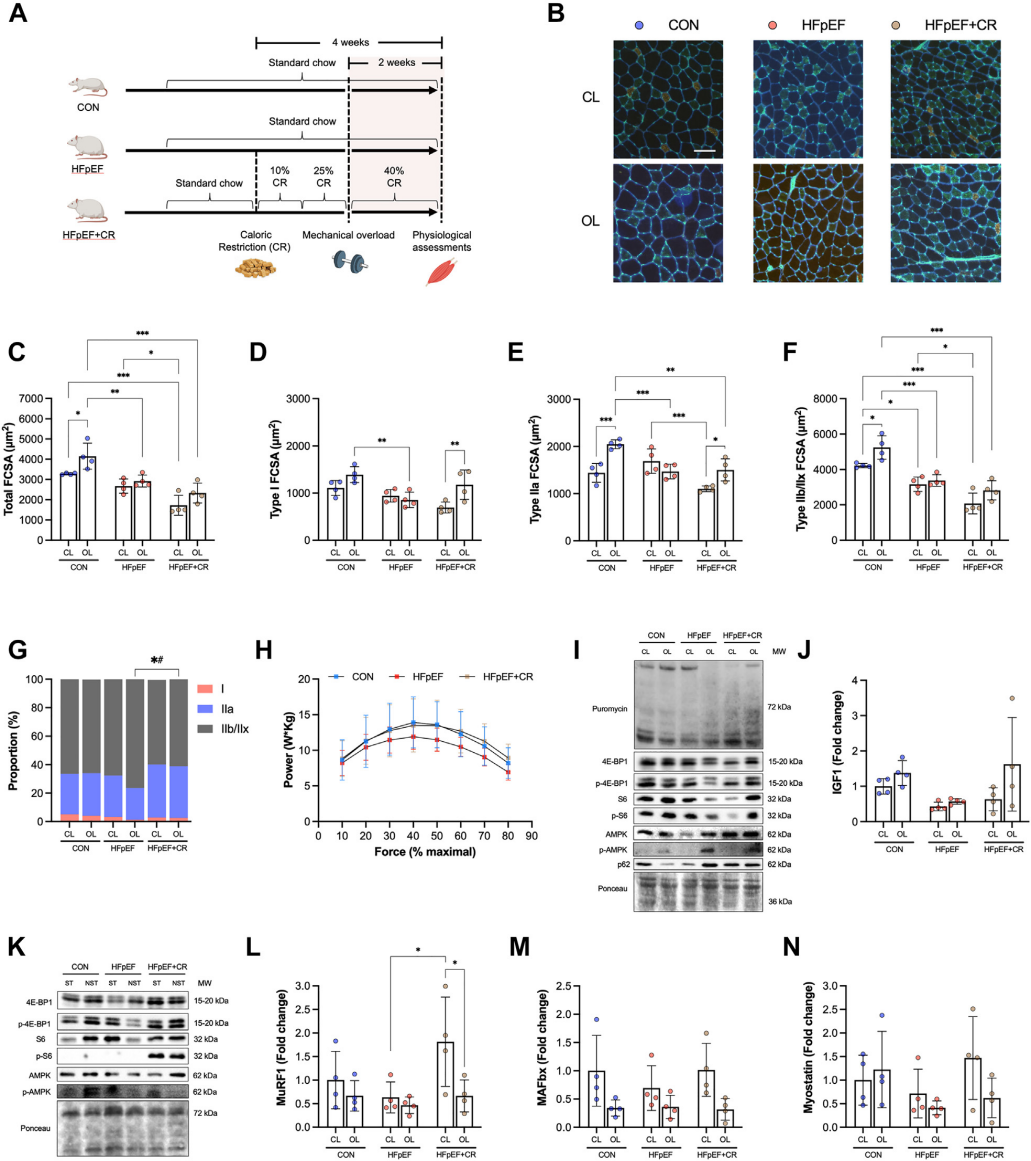
Acute Dietary Restriction Restores Myofiber Growth Following Mechanical Overload in HFpEF (A) Experimental design schematic showing start and endpoints of caloric restriction (CR) and mechanical overload of the EDL. (B) Representative images of contralateral (CL) and overloaded (OL) EDL muscles stained for Type I (red), Type IIa (green), and Type IIb/IIx (unstained/black) fibers and capillaries (bright green). (C) Total FCSA, (D) Type I FCSA, (E) Type II FCSA, (F) Type IIb/IIx FCSA, and (G) fiber type distribution of CL (CON n = 4, HFpEF n = 4, HFpEF+CR n = 4) and OL (CON n = 4, HFpEF n = 4, HFpEF+CR n = 4) EDL muscles. (H) Soleus contractile power measured across various percentages of maximal force (CON n = 4, HFpEF n = 4, HFpEF+CR n = 4). (I) EDL protein expression of puromycin, 4E-BP1, p-4E-BP1, S6, p-S6, AMPK, p-AMPK, and p62 in OL and CL muscles (CON n = 4, HFpEF n = 4, HFpEF+CR n = 4) and (J) gene expression for IGF1 (CON n = 4, HFpEF n = 4, HFpEF+CR n = 4). (K) Protein contents of 4E-BP1, p-4E-BP1, S6, p-S6, AMPK, and p-AMPK in stimulated (ST) and nonstimulated (NST) soleus. Gene expression of (L) MuRF1, (M) MAFbx, and (N) myostatin in CL and OL EDL muscles (CON n = 4, HFpEF n = 4, HFpEF+CR n = 4). Differences were assessed by 2-way analysis of variance followed by Bonferroni post hoc test. Data are presented as mean ± SD, and the level of significance was accepted as ∗*P* < 0.05; ∗∗*P* < 0.01; and ∗∗∗*P* < 0.001 for all analyses. Abbreviations as in Figure 1.

To test this hypothesis, we subjected male HFpEF rats to acute CR over 4 weeks^49^ starting with a step-wise reduction in calories by 10% and 25% in weeks 1 and 2, followed by 40% in the final 2 weeks in combination with mechanical overload to stimulate EDL myofiber growth (Figure 3A). Body mass was not changed, but hyperglycemia was reduced, whereas histological analysis of cardiac cryosections to assess structural remodeling showed that ventricular hypertrophy tended to be lower in HFpEF rats after CR (Supplemental Figure 6). Consistent with our initial experiments (Figure 2), analysis of EDL cryosections (Figure 3B) confirmed myofiber growth following overload was absent in HFpEF (Figures 3B and 3C). Remarkably, however, treatment with CR restored the hypertrophic response in HFpEF toward control rats, with a 35% increase in myofiber size observed (Figure 3C). In particular, myofiber growth in control rats and HFpEF+CR treatment was generally prevalent across all fiber types in response to overload, however statistical significance was detected for Type I and Type IIa in the caloric restriction group, and for Type IIa and Type IIx in the control group (Figures 3D to 3F). Increased myofiber growth with CR was not explained by changes in fiber type (Figure 3G) or capillarity (Supplemental Figures 7a to 7h).

To further investigate the specific effects of CR on skeletal muscle health in HFpEF, we performed detailed analyses across various muscles. Importantly, we did not find any negative effects of acute CR treatment such as overt muscle atrophy. Muscle mass for tibialis anterior, EDL, and soleus were not different between HFpEF rats treated with or without CR (Supplemental Figures 7i to 7q). We next performed in-depth functional assays on isolated soleus and found in vitro peak contractile power (the product of shortening velocity and force) tended to be 15% lower in HFpEF compared with control rats. By contrast, peak power showed an improvement following CR treatment, possibly due to beneficial effects on peak shortening velocity (Figure 3H, Supplemental Figures 8f to 8g). Furthermore, whereas soleus fiber size increased by 11% in HFpEF rats treated with compared to without CR, this did not reach statistical significance (*P* > 0.05) (Supplemental Figures 8i and 8j). Whereas our acute intervention did not cause a fiber type shift in HFpEF (Supplemental Figure 8k), we found a strong effect to increase capillarity (Supplemental Figures 8l to 8o). Together, these experiments suggest CR in HFpEF rejuvenates overload-induced myofiber growth, while having positive effects on basal muscle function and structure.

### Limitations to protein synthesis are not associated with decreased load-induced myofiber growth in HFpEF

We next set out to identify the mechanisms driving decreased load-induced myofiber growth in HFpEF and how CR could overcome this. Myofiber growth is primarily determined by elevated protein synthesis rates mainly via Akt-mTORC1 signaling to increase translational efficiency and capacity.^13^ To assess protein synthesis, we injected rats before sacrifice with puromycin, a structural analog of tyrosyl-tRNA that incorporates into nascent polypeptides.^50^ We then assessed global rates of protein synthesis by Western blotting using specific antibody against puromycin in EDL homogenates. We found no change in puromycin expression between groups when assessing relative changes between nonoverload vs overload muscle (Figure 3I, Supplemental Figure 9a). Decreased Akt-mTORC1 signaling is linked to reduced mechanical overload-induced myofiber hypertrophy in aging rats but restored by lifelong CR.^48^ We therefore probed phosphorylation of downstream mTORC1 targets S6 and 4E-BP1, but again found no difference between control, HFpEF, and HFpEF+CR rats in the nonoverload or overload conditions (Figure 3I, Supplemental Figures 9b to 9g). We also measured gene expression of IGF1, given it is a key upstream regulator of Akt-mTORC1 signaling, but this was not different between groups despite a trend for the response to be lower after overload in HFpEF (Figure 3J). Because changes in anabolic signaling can be transient, occurring over minutes to hours,^51^ we subjected the soleus muscle from healthy, HFpEF, and HFpEF+CR rats to repeated isometric contractions in vitro. After the protocol, we immediately froze tissue for Western blot analysis in order to measure acute expression of anabolic signaling proteins. However, we found no major differences between groups in terms of phosphorylated and total S6, 4E-BP1, and AMPK protein expression (Figure 3K, Supplemental Figures 9l to 9t).

Myofiber size is determined, not only by the rates of protein synthesis, but also by the relative rate of protein degradation.^52^ We therefore explored whether low myofiber growth in HFpEF was due to elevated catabolic signaling and increased atrogene expression. Activation of the energetic stress sensor AMPK is an upstream trigger that increases fiber atrophy in a FOXO-dependent manner,^52^ and has been linked to impaired load-induced myofiber growth.^53^ We found no differences between groups in phosphorylated AMPK following mechanical load challenge (Figure 3I, Supplemental Figures 9h to 9j). In line with this, no change was found in the expression of key ubiquitin proteasome-dependent atrogenes MuRF1 and MAFBx (Figures 3L and 3M), in the myostatin-TGFβ signaling pathway (Figure 3N) or autophagy-dependent p62 (Figure 3I, Supplemental Figure 9k), with all tending to be lower across groups after mechanical overload. Taken together, these data do not support inhibition of protein synthesis via Akt-mTORC1 signaling as a primary mechanism for decreased load-induced myofiber growth in HFpEF.

### Low myonuclear accretion as a mechanism of decreased myofiber growth in HFpEF

**Figure 4:**
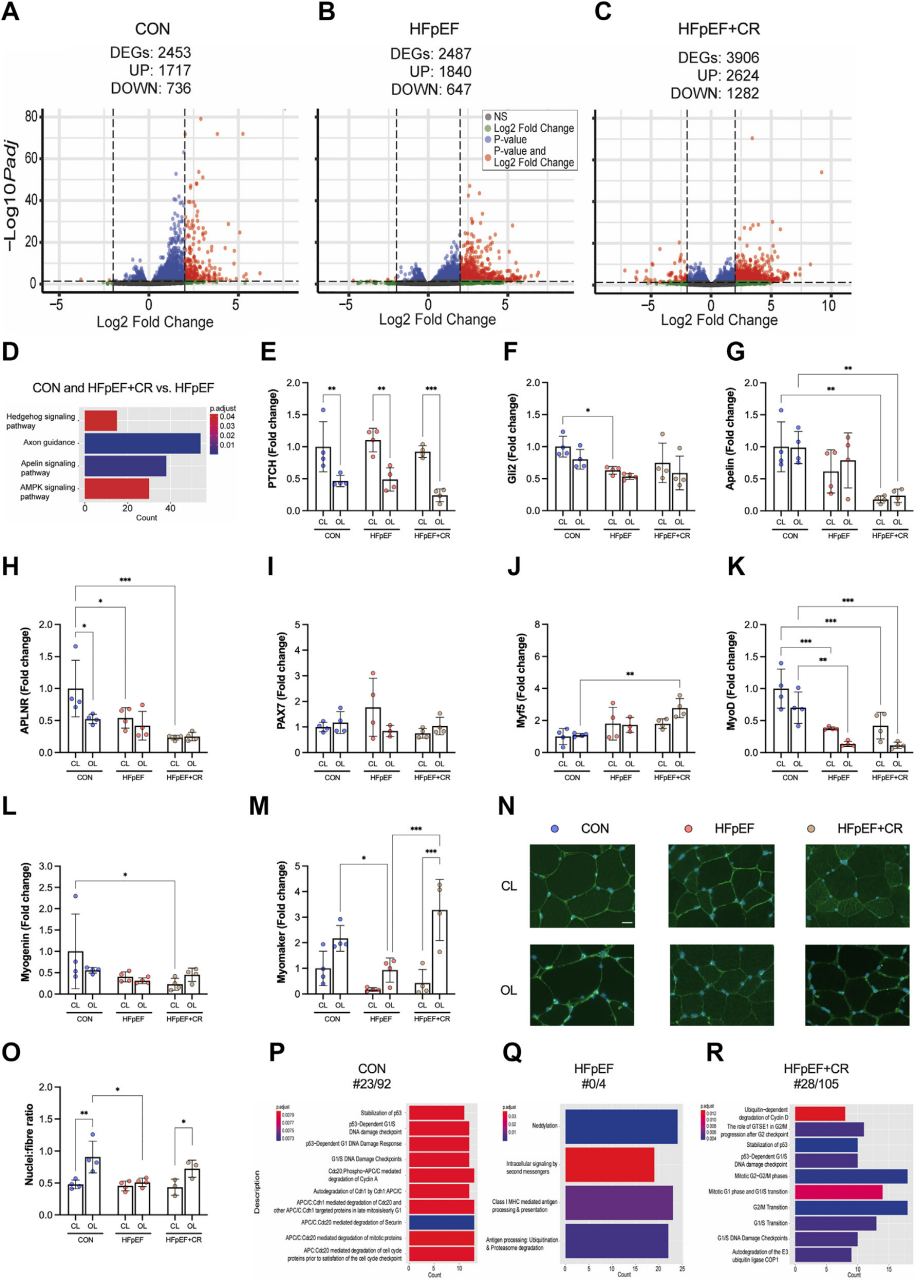
Caloric Restriction Increases Myonuclear Accretion in HFpEF Following Mechanical Overload Alongside Changes in the Myogenic Transcriptome Differentially expressed genes (DEGs) based on adjusted *P* value <0.05 in EDL muscles (contralateral [CL] vs overloaded [OL]) from (A) control (CON) (n = 4), (B) HFpEF (n = 4), and (C) HFpEF+CR (n = 4) rats. (D) Common KEGG pathway signature between control rats (n = 4) and HFpEF+CR (n = 4) vs HFpEF (n = 4). Gene expression of (E) PTCH, (F) Gli2, (G) apelin, (H) APLNR, (I) PAX7, (J) Myf5, (K) MyoD, (L) myogenin, and (M) myomaker in CL and OL muscles (CON n = 4, HFpEF n = 4, HFpEF+CR n = 4). (N) Representative images of CL and OL EDL muscles stained for nuclei (blue). (O) Number of myonuclei per fiber were determined (CON n = 4, HFpEF n = 4, HFpEF+CR n = 3). REACTOME pathway analysis showed clear differences in down-regulated pathways (especially the proportion of terms related to cell cycle regulation relative to the total number) between (P) CON (n = 4) vs (Q) HFpEF (n = 4), but not CON vs (R) HFpEF+CR (n = 4). The adjusted *P* value was corrected using the Benjamini and Hochberg method. Differences were assessed by 2-way analysis of variance followed by Bonferroni post hoc test. Data are presented as mean ± SD, and the level of significance was accepted as ∗*P* < 0.05; ∗∗*P* < 0.01; and ∗∗∗*P* < 0.001 for all analyses. Abbreviations as in Figure 1.

To explore additional signaling pathways regulating myofiber growth in HFpEF, we performed global transcriptomic profiling of EDL from healthy, HFpEF, and HFpEF+CR rats (comparing nonoverload and overload). Our analysis identified a large number of differentially expressed genes (adjusted *P* < 0.05) between EDL nonoverload and overload, with CR treatment resulting in the largest effect (Figures 4A to 4C). To provide further insight, we then performed KEGG pathway analysis which yielded 82, 92, and 100 up-regulated and 20, 17, and 35 down-regulated pathways in control, HFpEF, and HFpEF+CR rats between the EDL nonoverload vs overload (Supplemental Figures 10a to 10f). Therefore, we identified common KEGG pathway signatures between control and HFpEF+CR rats that were not present in HFpEF. This approach identified 4 unique pathways including Hedgehog, apelin, and AMPK signaling as well as axon guidance as common pathways in both control and HFpEF+CR rats, but not HFpEF (Figure 4D). AMPK is known to decrease mTORC1 activation and overload-induced myofiber hypertrophy,^53^ but, based upon our earlier observation of limited changes to protein synthesis and AMPK expression (Figure 3I), we focused our attention on the other pathways identified.

Our pathway analysis suggested decreased overload-induced myofiber growth in HFpEF could be associated with changes in MuSC homeostasis, given that Hedgehog (Hh) signaling is critical for myogenesis^54^ and linked to overload-induced myofiber growth,^55^ and apelin is a peptide myokine linked to sarcopenia that regulates MuSC function.^56^ We validated these targets and other relevant myogenic genes using quantitative polymerase chain reaction. For Hh signaling, we probed the expression of its receptor, patched (Ptch), and transcription factor, Gli2. Whereas Ptch was not different between groups, expression of Gli2 was lower in HFpEF compared to controls rats (Figures 4E and 4F). We also measured both apelin and its receptor but found no differences between groups (Figures 4G and 4H). Myofiber growth depends on the activation, proliferation, differentiation, and fusion of MuSCs.^57^ Therefore, we measured gene expression of myogenic transcription factors expressed by MuSCs (Pax7, Myf5, MyoD, myogenin) as well as the fusion protein myomaker in the overload and nonoverload EDL from control, HFpEF, and HFpEF+CR rats. Whereas this analysis did not find obvious differences between groups in terms of Pax7, Myf5, MyoD, and myogenin (Figures 4I to 4L), expression of myomaker was increased in overload EDL of healthy control and HFpEF+CR rats compared with HFpEF (Figure 4M). This indicates potential disruptions in MuSC-dependent myonuclear fusion could be present in HFpEF.^58^

The addition of new myonuclei via MuSCs is required for effective overload-induced myofiber growth.^40^^,^^58^^,^^59^ We therefore hypothesized addition of new myonuclei could be a limiting mechanism for load-induced myofiber growth in HFpEF, but can be overcome after acute CR given the known benefits on MuSC homeostasis.^23^ To test this and provide an index of myonuclear accretion, we quantified nuclei number per myofiber in EDL from overload and nonoverload cryosections in control, HFpEF, and HFpEF+CR rats (Figure 4N). Whereas myonuclear accretion showed a robust increase after overload in healthy control rats by 90%, this effect was blunted in HFpEF and rescued in HFpEF rats treated with CR (Figure 4O). These data indicate myofiber growth in HFpEF is limited, at least in part, by an inability to stimulate myonuclear accretion, most likely explained by impaired myogenesis. To probe for additional mechanisms related to perturbed myonuclear accretion, we performed further pathway analysis on our RNAseq data using KEGG and REACTOME. Generally, both databases gave concordant results. However, the annotations were significantly different for cell cycle regulators. In our analysis, REACTOME shows strong enrichment for genes related to cell cycle regulation, but not in KEGG. This approach identified a similar number of up-regulated pathways of ∼100 (Supplemental Figures 10g to 10i), but clear differences in down-regulated pathways were found between groups: only 4 in HFpEF, whereas there were ∼100 in control and ∼100 in HFpEF+CR rats (Figures 4P to 4R). Pathway interrogation revealed many terms related to cell cycle regulation involving G1/S transition, cyclins, mitotic DNA damage checkpoints, and stabilization of p53, with approximately 30 of the 100 terms found in control and HFpEF+CR rats, but none in HFpEF (Figures 4P to 4R). These data support our finding of myonuclear accretion as a limitation to myofiber growth in HFpEF and suggest this may be due to deficits in cell cycle regulation and perturbed MuSC homeostasis.^60^ Overall, our findings indicate that myonuclear accretion does not increase following overload-induced myofiber growth in HFpEF, perhaps explained by myogenic defects, but is restored after acute CR consistent with myofiber growth.

### Basal myogenic expression is dysregulated in rats and patients with HFpEF

**Figure 5:**
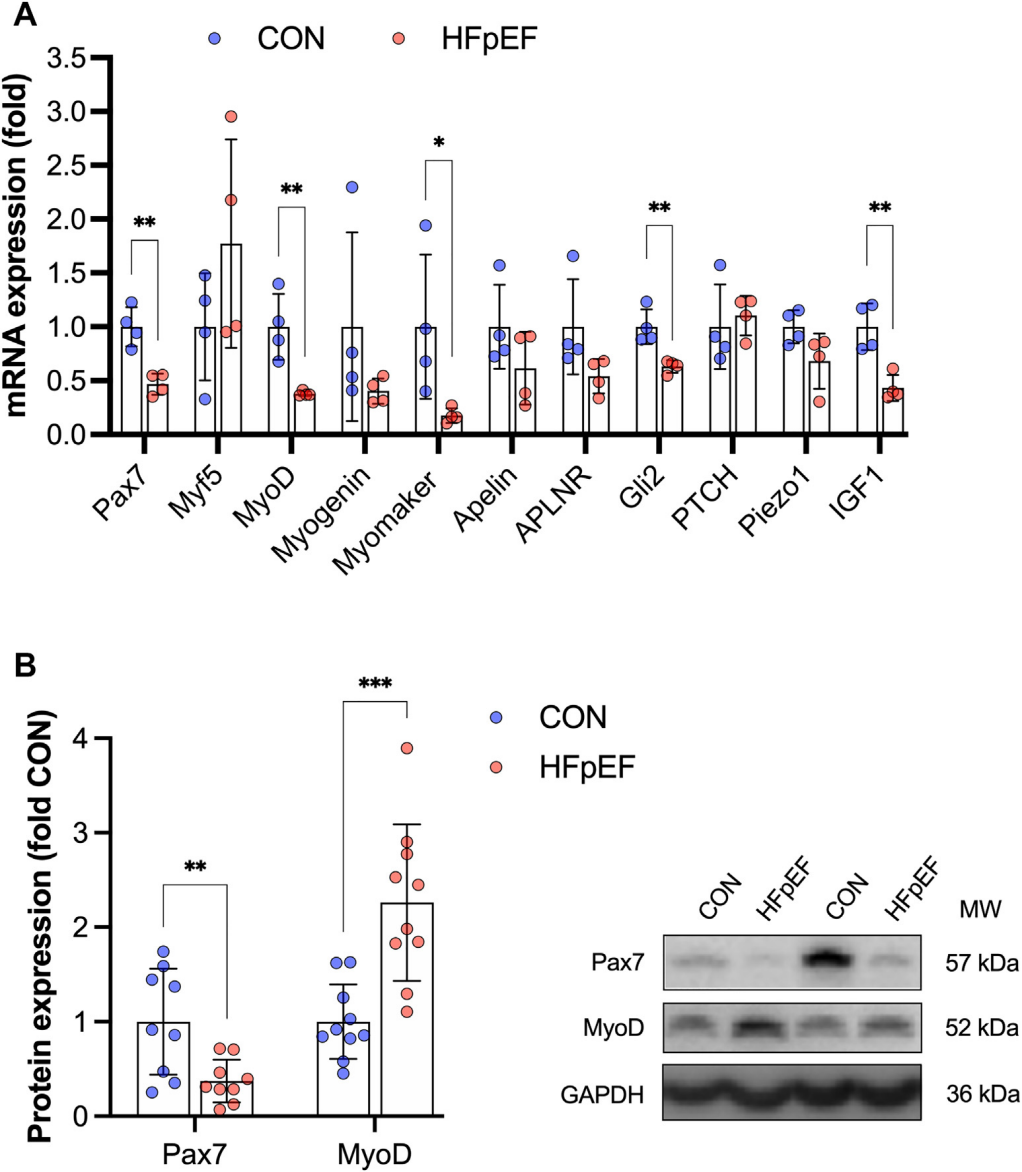
Basal Myogenic Expression Is Dysregulated in Rats and Patients With HFpEF Skeletal muscle gene expression of PAX7, Myf5, MyoD, myogenin, myomaker, apelin, APLNR, Gli2, PTCH, piezo1, and IGF1 measures in (A) rat EDL (CON n = 4, HFpEF n = 4) and (B) vastus lateralis biopsies from healthy age-matched control subjects (CON n = 10) and patients with diagnosed HFpEF (n = 10) measuring protein expression of Pax7 and MyoD normalized to loading control (GAPDH) with representative blots presented. Differences between groups were analyzed by unpaired 2-tailed Student *t*-tests. Data are presented as mean ± SD, and the level of significance was accepted as ∗*P* < 0.05, ∗∗ *P*< 0.01, ∗∗∗*P* < 0.001 for all analyses. Abbreviations as in Figures 1 and 2.

We next aimed to identify whether mechanisms regulating myonuclear accretion are perturbed in clinical HFpEF, by analyzing a cohort of well-characterized patients and age-matched control subjects (Supplemental Table 1).^14^^,^^15^^,^^18^ We measured basal myogenic expression in the vastus lateralis of patients with HFpEF compared with age-matched control subjects, and performed comparative measures in the EDL of our rat model using immunoblotting (Supplemental Table 2) and qPCR (Supplemental Table 3). Notably, rats with HFpEF showed lower basal expression in the key myogenic transcription factors Pax7 and MyoD by ∼50%, as well as the fusogen protein myomaker, when compared with control subjects (Figure 5A). We further measured established regulators of MuSC homeostasis including apelin^56^ and piezo1,^61^ but found only Hh signaling and IGF1 were significantly reduced in HFpEF vs control subjects (Figure 5A). Based on these findings, in our human samples, we focused on measuring basal expression of the myogenic transcription factors Pax7 and MyoD. Relative to healthy control subjects, we found protein expression of Pax7 was lower by ∼50% in patients with HFpEF, whereas myogenin was higher by 2-fold (Figure 5B). These data support that patients with HFpEF may have disturbances in basal MuSC homeostasis that could limit myofiber growth when subjected to physical loads.

## Discussion

HFpEF is no longer considered a simple syndrome of cardiac dysfunction, but rather a systemic disease that includes several extracardiac pathologies including skeletal muscle dysfunction.^2^^,^^4^ The skeletal muscle pathology is associated with worse symptoms and quality of life in HFpEF,^3^ however, we still have a poor understanding of the mechanisms and treatments. In this study, using a clinically relevant rat model characterized by cardiac dysfunction and exercise intolerance with multiple comorbidities including obesity, hypertension, and diabetes,^24^, ^25^, ^26^, ^27^, ^28^, ^29^, ^30^ we demonstrated a fundamental limitation for myofibers to hypertrophy in HFpEF when subjected to a resistance exercise intervention. We identified low myonuclear accretion as a mechanism for the decreased overload-induced myofiber growth in HFpEF, which was underpinned by changes in the transcriptional phenotype of myogenic homeostasis. These effects were reflected in skeletal muscle of patients with HFpEF. We established that skeletal muscle homeostasis could be rescued by acute dietary CR, which increased myonuclear accretion in line with myofiber growth. Furthermore, we highlighted that upstream limitations in muscle blood flow and capillarity are unlikely to impair overload-induced myofiber growth in HFpEF, although a role for mitochondrial dysfunction cannot be excluded. We also confirmed that treating HFpEF with pharmacological drugs to increase cardiac function did not rescue the skeletal muscle pathology.

HFpEF is associated with a multiple skeletal muscle abnormalities, including loss of muscle mass and strength, which are closely related to poor quality of life.^3^^,^^7^ One common approach is to use cardiac-targeted pharmacological therapeutics to increase cardiac function, with the expectation this will improve skeletal muscle perfusion and, subsequently, skeletal muscle pathology in heart failure. However consistent with past evidence,^62^^,^^63^ our findings suggest that cardiocentric medications have limited impact on skeletal muscle remodeling in HFpEF. These data are consistent with the majority of trials using pharmacological treatments in patients with HFpEF, which have shown neutral effects in terms of quality of life and clinical outcomes,^2^ excluding recent breakthrough using SGLT2 inhibitors.^64^ Together, this indicates that medications typically used to improve cardiac function and clinical outcomes in patients with HFpEF do not treat the skeletal muscle pathology. As such, identifying alternative nonpharmacological therapies for HFpEF skeletal muscle pathology remains an urgent priority.

One of the few effective treatments for HFpEF is exercise training, which improves physical function and quality of life.^19^ In addition, a landmark clinical trial showed that 20 weeks of CR in obese patients with HFpEF improved cardiac function, exercise capacity, glucose metabolism, and body mass, and subsequently, increased quality of life.^5^ Importantly, the application of CR in combination with exercise training showed additive benefits in these patients, including on skeletal muscle function.^5^^,^^20^ This guided us to explore whether CR alongside mechanical overload (ie, as a resistance exercise intervention) could stimulate therapeutic improvements in muscle mass and function. Low muscle mass is determined by the balance between myofiber anabolic and catabolic signaling.^52^ In studies on rats and humans with HFpEF, a general trend for increased catabolic signaling via ubiquitin proteasome-dependent activity has been reported.^14^^,^^15^^,^^18^ To date, however, the regulation of myofiber growth in HFpEF and associated anabolic signaling remained poorly defined. Strikingly, we identified that load-induced myofiber growth was severely attenuated in HFpEF compared with healthy control subjects. However, acute CR restored myofiber growth in HFpEF following mechanical loading. Myofiber growth is regulated via elevated protein synthesis and/or addition of new myonuclei via MuSCs.^13^ Evidence regarding skeletal muscle anabolic signaling/protein synthesis in HFpEF is absent, although in heart failure with reduced ejection fraction, some, but limited, data indicate protein synthesis is lower.^65^ Our findings do not support a role for impaired protein synthesis as a mechanism for decreased load-induced myofiber growth in HFpEF. This is in contrast to studies in aging and other conditions, which demonstrated that myofiber growth during mechanical load is reduced and limited by protein synthesis in an mTORC1-dependent manner,^66^^,^^67^ yet overcome by lifelong CR.^48^

Overload-induced myofiber growth can also be limited by myonuclear accretion.^68^ The addition of new myonuclei is governed by MuSCs, which reside in the basal membrane and are required to activate, proliferate, differentiate, and fuse into the myofiber in order to aid effective growth (with a proportion also returning to quiescence to maintain the MuSC pool).^57^ Evidence strongly supports that MuSC-dependent myonuclei accretion, including effective fusion via myomaker, is required for myofiber growth during increased physical loads.^40^^,^^58^^,^^59^ Our finding that CR increased myonuclear accretion along with myofiber growth supports a novel concept that MuSC-dependent myonuclear accretion is an important mechanism regulating skeletal muscle hypertrophy in HFpEF. Based on past evidence in other conditions,^23^ the mechanism(s) limiting myonuclear accretion and how this is overcome following acute CR in HFpEF is likely explained by changes in the MuSC environment, potentially due to deficits in cell cycle regulation (eg, via perturbing quiescence/activation)^60^ and/or myogenic progression/fusion (via MyoD, myomaker).^58^ This would align with past studies in aging showing MuSC dysfunction, senescence, and impaired cell cycle regulation are linked to sarcopenia,^69^ whereas use of CR can reverse these effects.^23^ MuSC function in HFpEF remains unknown, although a recent study in patients following aerobic exercise training showed relatively minor changes.^70^ This supports our hypothesis that patients with HFpEF have impaired MuSC homeostasis, which decreases skeletal muscle remodeling in response to adequate cues. Our findings of dysregulated basal myogenic expression in both HFpEF patients and rats further supports this hypothesis. Future studies are warranted to expand our knowledge of the role of MuSCs and their influence on the skeletal muscle pathology in HFpEF.

Effective myofiber growth also requires the integration of important upstream mechanisms, which include normal vascular and mitochondrial function. These mechanisms are impaired in both patients and animal models of HFpEF,^8^^,^^10^^,^^11^^,^^33^ but their role in myofiber growth remained unexplored. Low muscle perfusion in HFpEF could blunt overload-induced protein accretion by limiting delivery of critical nutrients and oxygen (ie, amino acids/insulin^13^) or by activating AMPK due to low energetic state to limit Akt-mTORC1 signaling.^53^ Alternatively, impaired angiogenesis impacts myonuclear accretion to blunt myofiber growth during overload.^41^ However, our data show that both muscle blood flow and capillarity are well preserved in HFpEF after mechanical overload, ruling out a role for vascular dysfunction. By contrast, we found low myofiber growth in HFpEF was associated with reduced mitochondrial function. Mitochondrial dysfunction is linked not only to decreased protein synthesis,^45^ but also to perturbed MuSC homeostasis.^69^ Interestingly, CR can reverse mitochondrial dysfunction in aging MuSC to rejuvenate myofiber growth in sarcopenia and post injury.^23^ Together, our data suggest that impaired muscle overload-induced hypertrophy in HFpEF may be linked to mitochondrial, but not vascular dysfunction.

The discovery that myofiber growth is low during mechanical loading in HFpEF, but can be increased by acute dietary CR, could have important clinical implications. Exercise training forms a central tenant of cardiac rehabilitation and is a key form of secondary prevention in patients with HFpEF.^71^ Although it remains unclear whether myofiber growth in patients with HFpEF is blunted following sustained strength training, recent evidence indicates a low response in skeletal muscle remodeling following endurance training alone.^70^ Our findings suggest merging periods of acute CR with targeted strength training may optimize beneficial muscle adaptions, thus promoting faster and greater gains in functional performance and potential clinical outcomes. Beyond muscle mass, our data indicate CR could benefit intrinsic muscle function and muscle oxygen supply that may enhance exercise capacity and quality of life in patients.^5^ Overall, this indicates that cardiac rehabilitation could optimize benefits to skeletal muscle in patients with HFpEF that incorporate acute CR periods. This combination offers a novel and viable nonpharmacological treatment strategy to alleviate the skeletal muscle pathology, which is highly relevant given that many pharmacological approaches to treat HFpEF have been ineffective.^2^

### Study Limitations

The findings must be interpreted with caution, given many experiments were performed in an animal model of HFpEF and not directly in humans. For example, it should be noted that fiber types are not identical between rats and humans, meaning there are potential limitations when comparing species.^72^ However, our observations in human tissue aligned with our experimental animal data. Moreover, the ZSF1 obese rat model represents a well-established experimental model of HFpEF that closely reflects the patient phenotype.^16^^,^^18^^,^^24^, ^25^, ^26^, ^27^, ^28^, ^29^, ^30^ The present study included mostly female patients, and therefore, our findings may not fully translate to males with HFpEF, although current evidence indicates no apparent sex-specific skeletal muscle differences.^10^^,^^73^ It should be appreciated that our findings in patients came from a leg muscle (vastus lateralis) that may not translate to other muscles in the body. However, because muscle alterations in heart failure patients from the lower limb have been closely correlated to changes in the upper limb,^74^ it is likely our findings are important for other muscle groups. Further research will be required to confirm this suggestion. An inherent limitation for interpreting bulk skeletal muscle analysis is that fiber-type–specific changes cannot be discerned, which may have masked myofiber-specific changes in molecular signaling being detected.^75^ We also assessed a single time point after mechanical overload, therefore, further complexity in protein synthesis and/or myonuclear accretion may be present over a temporal range. However, we did assess the acute response immediately following muscle contraction and found no evidence to contradict our conclusions.

This study focused on the acute effects of CR. A limitation of this study was that cardiac function was not directly assessed after caloric restriction, although we did measure structural changes via histology. Given past studies in heart failure have documented caloric restriction increases cardiac function,^5^^,^^76^^,^^77^ and our current study confirmed beneficial effects on cardiac remodeling, together this indicates both function and structure were probably improved after caloric restriction in HFpEF. Our experiments were performed in male rats, and we cannot exclude sexual dimorphism in the effects of CR on the muscle phenotype in HFpEF. Moreover, the long-term effects of CR in HFpEF remain uncertain. Some heart failure patients are susceptible to sarcopenia and frailty,^12^ whereas obese patients in general show better survival than normal or underweight patients,^78^ meaning the clinical effects of CR on muscle loss must be carefully considered.^5^^,^^20^ We also acknowledge that to fully support clinical translation, a randomized exercise trial is required where patients with HFpEF and healthy control subjects perform strength training, and myonuclear accretion and myofiber growth are assessed, however, this was beyond the scope of the current study.

## Conclusions

This study has identified a novel mechanism to explain the skeletal muscle pathology in HFpEF and revealed an effective treatment centered on nonpharmacological approaches of combined diet and strength exercise.

Perspectives **COMPETENCY IN MEDICAL KNOWLEDGE:** Key symptoms in HFpEF such as exercise intolerance cannot be solely explained by cardiac dysfunction, meaning other treatment targets should be considered. HFpEF is known to induce a skeletal muscle pathology, which is closely linked to worse symptoms. What mechanisms cause and how to treat skeletal muscle pathology in HFpEF remain poorly known. This paper comprehensively addresses what mechanisms are involved in the skeletal muscle pathology in HFpEF and highlights therapeutic targets and relevant treatments in the form of nonpharmacological approaches related to exercise and diet that could be important for optimizing future treatment in the clinic.

**TRANSLATIONAL OUTLOOK:** We have identified potential novel mechanisms and treatments of skeletal muscle pathology in HFpEF using animal models, and show data these are conserved in patients. Given clinical evidence indicates that caloric restriction alone reduces symptoms in patients with HFpEF, tailored combination therapy that optimizes both exercise regimes and pharmacological treatments warrant further exploration as this will likely provide the greatest benefits to quality of life.

## Funding Support and Author Disclosures

Dr Espino-Gonzalez is a recipient of a doctoral fellowship from the Mexican National Council of Science and Technology (CONACYT). Dr Altara’s work was supported by a grant from the K.G. Jebsen Center for Heart Failure Research. Dr Cheng is supported by BHF Mautner Career Development Fellowship. Dr Justo da Silva was supported by the South-Eastern Norway Regional Health Authority (HSØ-RHF, Project No. 25674). Dr Booz has received support from the Pharmacology Clinical Research Core of the University of Mississippi Medical Center School of Medicine. Dr Bowen has received funding from the Medical Research Council (UK) (MR/S025472/1) and Heart Research UK (TRP16/19). All other authors have reported that they have no relationships relevant to the contents of this paper to disclose.
