# Supplementary material for: Caloric Restriction Rejuvenates Skeletal Muscle Growth in Heart Failure With Preserved Ejection Fraction
Source: JACC Basic Transl Sci. 2023 Dec 6;9(2):223–40. doi: 10.1016/j.jacbts.2023.09.014 (PMC10950401; doi:10.1016/j.jacbts.2023.09.014)
Supplement: Supplemental Appendix [file mmc1.pdf]

**Supplemental Appendix**


Supplemental Methods – P2

Supplemental Tables – P7

Supplemental Table 1 – P7

Supplemental Table 2 – P8

Supplemental Table 3 – P9

Supplemental Figures – P10

Supplemental Figure 1 – P10

Supplemental Figure 2 – P11

Supplemental Figure 3 – P12

Supplemental Figure 4 – P13

Supplemental Figure 5 – P14

Supplemental Figure 6 – P15

Supplemental Figure 7 – P16

Supplemental Figure 8 – P17

Supplemental Figure 9 – P18

Supplemental Figure 10 – P19

Supplemental References – P20

## Supplemental Methods

### Ethical approval

Procedures and experiments in rats treated with cardiovascular medications (i.e., Sac/Val) were performed at the University of Oslo, Norway, and approved by the Norwegian Food Safety Authority committee (Mattilsynet) for animal research (FOTS protocol number 15886) in accordance with the national regulations, the European Convention for the Protection of Vertebrate Animals used for Experimental and Other Scientific Purposes (ETS No.123), and the European Directive 2010/63/EU on the protection of animals used for scientific purposes. All other procedures and experiments were performed in accordance with the UK Scientific Procedures (Animals) Act 1986 and local approval was given by the University of Leeds Animal Welfare and Ethical Review Committee (70/08674). For the clinical study, all participants provided written informed consent, with research protocols approved by the University of Leipzig Ethics Committee[1].

### Animals

Male obese (HFpEF) and lean (controls) diabetic Zucker fatty/spontaneously hypertensive heart failure F1 hybrid (ZSF1) rats were purchased from Charles River at 13-18 weeks of age. This hybrid rat is a cross between a ZDF female and an SHHF male rat. Whereas both lean and obese ZSF1 rats inherit the hypertension gene, only the obese ZSF1 rats inherit a mutation in the leptin receptor gene ($Lepr^{fa}Lepr^{cp}/Crl$) that drives weight gain and associated metabolic impairments, with typical signs of HFpEF developing as early as 10 weeks of age[2] and well established after 20 weeks[2, 3, 4, 5, 6, 7, 8, 9, 10]. All rats were kept at a 12 h light/dark cycle. Unless stated, rats were fed *ad libitum* with standard chow and access to water.

### Study design

To evaluate the effects of cardiovascular medications (Sac/Val) in HFpEF skeletal muscle, obese male ZSF1 rats at 20 weeks of age were randomly assigned to the following groups: HFpEF+Vehicle (HFpEF+Veh; n=8) or HFpEF+Sac/Val (HFpEF+Sac/Val n=6); and compared to their respective lean controls (CON; n = 11). Sac/Val (68 mg/kg body mass/d) or vehicle was delivered via oral administration (gavage) for 10 weeks. In the second set of experiments, male lean (n=8) and obese (n=8) ZSF1 rats at 20 weeks of age underwent surgery to induce mechanical overload for 14 days of the extensor digitorum longus (EDL) muscle. In the third set of experiments, male lean (n=4), obese ZSF1 (n=4), and obese ZSF1 rats treated with dietary caloric restriction (HFpEF+CR) (n=4) were compared starting from 18 weeks of age, with overload induced at 20 weeks of age for 14 days. CR was initiated at week 1 at 10% restriction, increased to 25% restriction in week 2, and maintained at 40% restriction between weeks 3-4. The sample size for the CR intervention was determined using *a priori* power calculation based on the second set of experiments and a standard deviation (SD) for muscle size of 12% ($\alpha$ = 0.05, power = 80%, 30% effect; n = 4 per group).

### Cardiometabolic function

Cardiac function was assessed by transthoracic echocardiography using a VEVO 3100 high-resolution *in vivo* imaging system from VisualSonics. Briefly, animals were maintained under anesthesia (1.5-2% isoflurane mixed with 100% oxygen) on a pre-warmed ECG transducer pad with body temperature and ECG monitored. Measurements were made with an MS250 transducer, frequency set at 20 MHz. B-mode measurements in the parasternal long axis view were obtained to assess the function and dimension of the left ventricle (LV). M-mode tracings through the aortic root and the left atrium (LA) were used to assess LA diameter. LVEF was calculated as 100 * ((LV Vol;d - LV Vol;s) / LV Vol;d). LV mass was estimated by the formula: 1.053 * ((LVID;d + LVPW;d + IVS;d)3 - LVID;d3). Relative wall thickness (RWT) was calculated as 2 * LVPW;d / LVID;d.7 E and A waves in LV filling velocities were assessed *via* pulsed-wave Doppler in the parasternal long axis view. Early (E wave) and late (A wave) ventricular filling velocities were assessed *via* pulsed-wave Doppler in an apical 4-chamber view. Myocardial velocities (e' and A') were measured using tissue Doppler imaging at the level of the basal septal segment of the LV in an apical 4-chamber view. Cardiac output (CO) was

estimated from the dimension of the LV on the M-mode view. Cardiometabolic impairments were confirmed by measures of body weight, mean arterial pressure (*via* an implanted carotid catheter; PP10) with a blood pressure transducer (BP transducer, AD Instruments, UK) and fasting blood glucose levels (*via* a commercial blood glucose meter; FreeStyle Mini Meter).

**Mechanical overload**

To induce EDL hypertrophy, unilateral surgical ablation of the TA was performed as previously described[11]. Briefly, rats were weighed and maintained under isoflurane anesthesia (2-3% in 100% oxygen). Under aseptic conditions, the distal TA tendon was transected and the majority of the muscle belly separated using blunt dissection. The TA was removed near to its proximal insertion, while keeping the underlying EDL muscle intact. The contralateral limb was used for sham surgery, in which TA and EDL muscles were identified but not separated. Post-operative analgesia (buprenorphine; Vetergesic, Ceva, Amersham, UK; 0.05 mg/kg) and antibiotic (Enrofloxacin ("Baytril"); Bayer, Reading, UK; 2.5 mg/kg) were provided to all animals after surgery. All rats were ambulatory throughout the 14 day experimental period, and no postoperative complications were observed. The mechanical overload model allows a paired comparison between contralateral (non-overloaded) and overloaded muscles, which avoids biases resulting from the use of different animals[12].

***In situ* EDL muscle performance and femoral artery blood flow**

Function of the EDL muscle and femoral artery blood flow were evaluated *in situ* according to our published protocol[13]. Data from the relative control leg have been published previously[13]. Anesthesia was induced using isoflurane (4% in 100% oxygen) and maintained using a constant syringe pump infusion of (30-35 mg/kg/h) Alfaxalone (Jurox, Crawley, UK) *via* an implanted jugular vein catheter. EDL isometric forces were measured with a lever arm force transducer (305B-LR: Aurora Scientific, Aurora, ON, Canada). It was necessary in the sham surgery limb to remove the TA to provide unimpeded access to the underlying EDL. Optimal muscle length and supramaximal current delivery were determined by electric stimulation (1 Hz, 0.3 ms pulse width) *via* electrodes placed adjacent to the popliteal nerve[14]. Simultaneous bilateral femoral artery blood flow was measured using perivascular flow probes (0.7PSB; Transonic, Ithaca, NY, USA)[15]. PowerLab and LabChart software (AD Instruments, UK) was used to record all data. Blood flow is presented in absolute units (ml $min^{-1}$) and normalized to mean carotid pressure (vascular conductance: ml $min^{-1}$ mm $Hg^{-1}$).

EDL fatigue resistance and tetanic force were also quantified. Fatigue resistance was determined as the ratio of end-stimulation: peak isometric twitch force, as quantified over the course of a 3 min period of continuous 10 Hz stimulation. An average of 5 consecutive twitches was calculated for peak and end-stimulation forces. Following restoration of pre-fatigue resting blood flow (~10 min recovery), tetanic forces were quantified by 200 Hz stimulation (200 ms duration). Data is presented in absolute units (g) and normalized to wet muscle mass (g/mg EDL).

***In vitro* soleus functional assessment**

Left soleus were dissected to allow *in vitro* contractile function to be assessed using a length-controlled lever system (305C, Aurora Scientific, Aurora, Canada), as previously described[13]. Muscles were prepared in a Krebs–Henseleit solution (117 NaCl, 4.7 KCl, 1.2 $MgSO_4$, 1.2 $KH_2PO_4$, 24.8 $NaHCO_3$, 2.5 $CaCl_2$, 11.1 glucose; in mmol $l^{-1}$) at 4°C equilibrated with 95% $O_2$/5% $CO_2$. A muscle bundle was then mounted vertically in a buffer-filled organ bath (Krebs–Henseleit solution), set at optimal length ($L_o$), and after 15 min at ~21°C was stimulated according to two distinct protocols: I) isometric force-frequency, and ii) isotonic force-velocity. The force-frequency relationship was determined in response to stimulation at 1, 15, 30, 50, 80, 120 and 150 Hz, with 1 min of recovery between contractions. After a 5 min period during which muscle length was measured using digital calipers, the force-velocity relationship was determined via isotonic contractions (80-10% of the maximal tetanic force; each separated by 1 min) at 150 Hz for 300 ms. Shortening velocity ($L_o$/s) was determined 10 ms after the first

change in length and on the linear section of the transient (605A DMA software, Aurora Scientific). Force (N) was normalized to muscle cross-sectional area (CSA; $cm^2$) by dividing muscle mass (g) by the product of $L_o$ (cm) and estimated muscle density (1.06 $g/cm^3$), which allowed specific force in $N/cm^2$ to be calculated.

**Mitochondrial respiration**

*In situ* mitochondrial respiration ($JO_2$) was assessed in permeabilized EDL muscle fibers using high resolution respirometry (Oxygraph-2k: Oroboros Instruments, Innsbruck, Austria), as described elsewhere[16]. Samples were dissected in BIOPS solution, permeabilized in saponin (50 µg/ml) for 30 min, washed (twice) in MIR06 for 10 min, weighed and immediately transferred to the chambers of the high-resolution respirometer, where each chamber contained 2 mL of MiR05 at 37°C. Chambers were oxygenated (~450 $nmol \cdot mL^{-1}$) before starting the experiments. A standard protocol[16, 17] for substrate, uncoupler, and inhibitor titration (SUIT) was then used for measuring leak respiration with complex I substrates ($L_I$) and oxidative phosphorylation with complex I ($P_I$) and complex I+II substrates ($P_{I+II}$) as well as uncoupled respiration in the presence of complex I+II ($E_{I+II}$) and complex II substrates ($E_{II}$). Substrates were injected in the following order: blebbistain (2 µL), glutamate (10 µL), malate (2.5 µL), pyruvate (5 µL), ADP (10 µL), cytochrome c (5 µL), succinate (20 µL), FCCP (1 µL), rotenone (5 µL), antimycin A (5 µL), ascorbate (5 µL), TMPD (5 µL) and sodium azide (10 µL). Cytochrome c was added to evaluate the integrity of the mitochondrial outer membrane (samples with a >15% increase in respiration rate were excluded) and intrinsic function was assessed following normalization to mitochondrial content (complex IV activity; $C_{IV}$) in addition to the coupling efficiency (i.e., respiratory control ratio, RCR: complex I phosphorylated state/complex I leak respiration).

**Histological analysis**

Skeletal muscles were mounted in optimal cutting temperature embedding medium (Thermo Scientific, Loughborough, UK), frozen in liquid nitrogen-cooled isopentane and stored at -80°C. Muscle samples were cryosectioned (-20°C, 10 µm), mounted on polylysine-coated slides and stored at -20°C until staining. Muscle fiber type composition and capillary network morphology were determined as previously described[13]. Briefly, sections were fixed for 2 min in 2% paraformaldehyde, washed in phosphate-buffered saline (PBS; P4417, Sigma-Aldrich, St Louis, MO) and blocked for 10 min in 1% bovine serum albumin (A6003, Sigma-Aldrich, St Louis, MO). Myofiber boundaries were labelled with a rabbit anti-laminin antibody (1:200; L9393, Sigma-Aldrich, St Louis, MO), while monoclonal myosin heavy chain antibodies BA-D5 (IgG2B, 1:1000) and SC-71 (IgG1, 1:500) were used to label Type I (oxidative) and Type IIa (fast oxidative, glycolytic) fibers, respectively (Developmental Studies Hybridoma Bank, Iowa City, IA, USA). Unstained fibers were quantified as Type IIb/IIx. After washing in PBS, sections were incubated for 60 min with secondary antibodies Alexa Fluor 555 (conjugated goat anti-mouse IgG, 1:1000, A-21422, Thermo Fisher Scientific, Waltham, MA) and Alexa Fluor 488 (conjugated rabbit anti-mouse IgG, 1:1000, A11059, Thermo Fisher Scientific, Waltham, MA). Finally, capillaries were labelled with fluorescein-conjugated *Griffonia simplicifolia* lectin I (Vector Labs, Peterborough, UK; FL-1101), a carbohydrate-binding protein (lectin) specific to rodent endothelial cells. Slides were imaged at x10 magnification using a Nikon Eclipse E600 (Nikon, Tokyo, Japan) optical microscope attached to a digital camera (QIMAGING, MicroPublisher 5.0 RTV, Surrey, BC, Canada). Fiber type-specific cross-sectional area (FCSA), capillary-to-fiber ratio (C:F), capillary density (CD), capillary domain area (CDA), local capillary-to-fiber ratio (LCFR), and local capillary density (LCD) were derived from histological sections using DTect software in MATLAB (The MathWorks, Cambridge, United Kingdom)[18]. Two regions of interest (∼155 fibers) were taken at the core of the sections to establish an unbiased counting frame, taking into account the regional heterogeneity across muscles[13].

To investigate the functional consequence of the capillary network morphology we mathematically modelled skeletal muscle oxygen transport kinetics using a custom MATLAB

oxygen transport modeler (OTM), as described in more detail in our previous studies[13, 18]. Using digitized images of EDL muscle cryosections, the OTM incorporates fiber type-specific features (i.e., FCSA, fiber type distribution and capillary locations) and estimates of capillary radius (1.8-2.5 x 10-$^{4}$ cm), muscle oxygen consumption (15.7 x $10^{-5}$ ml $O_2$ $ml^{-1}$ $s^{-1}$), myoglobin concentration (10.2 x $10^{-3}$ ml $O_2$ $ml^{-1}$), $O_2$ solubility (3.89 x 10-5 ml $O_2$ $ml^{-1}$ $mmHg^{-1}$) and diffusivity (1.73 x $10^{-7}$ $cm^2$ $s^{-1}$) to model local oxygen consumption and estimate tissue oxygen partial pressure ($PO_2$) distribution.

To characterize muscle fibrosis, soleus and EDL cryosections were stained with Sirius red (Sigma-Aldrich, St Louis, MO, USA). Briefly, sections (10 µm thickness) were hydrated with distilled water, incubated with Picro-Sirius Red (1 h), rinsed in acetic acid solution (0.5%), and dehydrated in absolute alcohol. The relative area of the sections occupied by Sirius red staining was then calculated using ImageJ software. A similar approach was also used to characterize cardiac remodeling in rats treated with or without caloric restriction, with left and right ventricular thickness subsequently determined.

EDL cryosections (10 µm thickness) were stained with DAPI to quantify the number of nuclei per fiber. Sections were fixed for 2 minutes in 2% paraformaldehyde, washed in PBS (P4417, Sigma-Aldrich, St Louis, MO) and blocked for 10 minutes in 1% BSA (A6003, Sigma-Aldrich, St Louis, MO). Muscle fiber boundaries were labelled with a rabbit anti-laminin antibody (1:200; L9393, Sigma-Aldrich, St Louis, MO). Sections were then incubated for 60 min with Alexa Fluor 488 (conjugated rabbit anti-mouse IgG, 1:1000, A11059, Thermo Fisher Scientific, Waltham, MA) and mounted in slides using mounting medium with DAPI. Three washes with PBS were performed between each step. Myonuclei and fibers were manually counted in images using ImageJ software. A myonucleus was defined if it met one of the following criteria: 1) it was clearly located within the myofiber boundary; 2) it was on the boundary facing inside the fiber; or 3) >50% of the area fell inside the fiber boundary[19].

**Protein extraction and western blot analysis**
Frozen muscle samples were homogenized in RIPA buffer (50 mM Tris, 150 mM sodium chloride, 1 mM EDTA, 1% NP-40, 0.25% sodium-deoxycholate, 0.1% SDS, 1% Triton X-100; pH 7.4) containing a protease and phosphatase inhibitor cocktail (Thermo Scientific A32961), sonicated, and centrifuged at 13,000 rpm for 10 min. The supernatant was collected, and protein content was quantified *via* BCA assay (Thermo Scientific 23225). Muscle homogenates with equal amounts of protein (20 µg) were mixed with loading buffer (126 mmol/L Tris-HCl, 20% glycerol, 4% SDS, 1.0% 2-mercaptoethanol, 0.005% bromophenol blue; pH 6.8), and separated by electrophoresis (1.5 hours at 90 V) on 8%-12% sodium dodecyl sulfate polyacrylamide gels and transferred to a nitrocellulose membrane (Amersham™ Protran® GE10600003). Membranes were stained with Ponceau S to determine total protein content and rinsed with Tris-buffered saline/Tween solution (0.5 M NaCl; 50 mM Tris–HCl, pH 7.4; and 0.1% Tween 20). Membranes were then blocked with 5% milk or 5% BSA, and incubated overnight at 4°C with primary antibodies (see Supplementary Table 2). After a 5-min wash (3x) in Tris-buffered saline/Tween solution, membranes were incubated with secondary antibodies (Supplementary Table 2) for 1 h at room temperature. Membranes were again washed for 5 min (3x) in Tris-buffered saline/Tween solution, and labelled proteins were detected using an enhanced chemiluminescence system (iBright750, Invitrogen by Thermo Fisher Scientific CL750) and densitometry quantified using ImageJ software (Scion Corp., National Institutes of Health, Bethesda, MD, USA). Protein expression was normalized to total protein content (Ponceau S) and presented as fold change to unloaded muscle from controls.

**Puromycin assay**
The SUnSET method was used to measure protein synthesis[20]. Briefly, 30 min prior to euthanasia animals received an i.p. injection of puromycin (0.040 µmol/g body mass;

dissolved in PBS), which was then detected in homogenized muscle samples via Western blotting, as described previously[20].

**RNA isolation and real-time PCR**

RNA extraction of the EDL was performed using the Trizol-chloroform-isopropanol method following Trizol reagent solution user guide (Thermo Fisher Scientific, Cat No. AM9738). Briefly, approximately 10 mg of tissue was homogenized in a tissue lyser (Qiagen TissueLyser II, Cat No. 85300) with 1 ml Trizol reagent solution and a glass bead. Subsequently, 200 µl chloroform was added, shaken and incubated at room temperature for 5 minutes and centrifuged (12,000rpm, 4°C, 15 minutes) to separate the RNA containing phase. The RNA containing phase was combined with 500 µl isopropanol, vortexed and left to incubate at room temperature for 10 minutes and then centrifuged to produce an RNA pellet (12,000rpm, 4°C, 15 minutes). The Pellet was then washed twice in 75% ethanol, centrifuged (12,000rpm, 4°C, 5 minutes) and left to dry for 1 hour. Once dried 30 µl of RNase/DNase free water was added to the pellet. RNA purification using the RNA clean and concentrate-5 kit was conducted following the kit protocol (Zymo Research, Cat No. R1013) resulting in high quality RNA in 20 µl of RNase free water confirmed using a nanodrop. cDNA synthesis was conducted using the $RT^2$ First Strand kit (Qiagen, Cat No. 330404) following kit protocols. PCR was performed using SYBR green Light Cycler 480 I master mix (Roche, Cat No. 04887352001), and a Bio-Rad CFX 96 Thermocycler. Genes were run in triplicate for each sample using lab validated primers containing both forward and reverse primers (Supplementary Table 2). Delta-Delta-CT method was then performed to calculate fold expression changes normalized to beta actin.

**RNA sequencing**

Total RNA was extracted using the phenol-chloroform extraction method. There were three conditions; control, HFpEF, and HFpEF+CR with four biological replicates from each leg (left and right). Novogene, Cambridge, United Kingdom, performed RNA sequencing (RNA-seq). The library preparation was constructed using the TruSeq Stranded mRNA kit (Illumina), following the manufacturer's recommended protocol. The libraries were sequenced on the HiSeq 4000 platform with 150 bp paired-end strategy. On average, 84.59 M high-quality reads were generated from the RNA sequencing project. The 24 raw reads were uploaded to the ENA-EMBL-EBI database under the accession number E-MTAB-12494.

**RNA sequencing data analysis**

STAR aligner[21] was used to map the raw reads to the *Rattus norvegicus* reference genome (Ensembl mRatBN7.2). The gene expression levels were quantified by featureCounts[21, 22] and normalized by DESeq2[23] using the negative binomial model. Differentially expressed genes were defined based on adjusted p-value < 0.05. For pathway enrichment analyses, Kyoto Encyclopedia of Genes and Genomes (KEGG) and REACTOME databases were searched via clusterProfiler and ReactomePA, respectively, to predict potential enriched pathways. The significant terms were selected based on adjusted p-value < 0.05. The adjusted p-value was corrected based on Benjamini and Hochberg method. Volcano and dot plots were generated using the GGPlot2 function in R Statistical Software (v4.3.0; R Core Team 2022)[24].

**Human experiments**

For human studies, age-matched healthy controls (n=10) and patients with diagnosed HFpEF (n=10) were included. Clinical characteristics of these patients have been previously published[1], however due to limited muscle tissue availability a smaller sample size is presented in this study. In brief, a *vastus lateralis* muscle sample was excised under local anesthesia via a percutaneous needle, as detailed elsewhere[1]. The tissue sample was cleaned free of connective tissue and immediately snap frozen in liquid nitrogen for subsequent western blotting as described above, although protein expression was normalized to loading control GAPDH.

## Supplemental Tables

**Supplemental Table 1:** Human characteristics from age-matched healthy people compared to patients with HFpEF

| | Healthy (n=10) | HFpEF (n=10) | p-value |
|---|---|---|---|
| **Age [years]** | 68.9 ± 2.3 | 72.4 ± 1.2 | 0.12 |
| **Female gender [n] (%)** | 7 (70.0) | 8 (80.0) | 0.95 |
| **BMI [kg/m²]** | 29.0 ± 1.1 | 31. 7 ± 1.8 | 0.22 |
| **Systolic blood pressure [mmHg]** | 133.0 ± 1.7 | 136.1 ± 2.8 | 0.45 |
| **NT-proBNP [pg/ml]** | 74 ± 2 | 1072 ± 329 | **<0.001** |
| **LVEF [%]** | 65.1 ± 2.1 | 61.6 ± 1.9 | 0.44 |
| **E/e´ septal** | 8.27 ± 0.9 | 17.3 ± 2.3 | **<0.001** |
| **E/e´ lateral** | 6.30 ± 0.4 | 11.4 ± 1.1 | **<0.001** |

Data are presented as mean±SD

**Supplemental Table 2.** List of antibodies

| **Primary antibodies** | **Dilution** | **Company** |
|---|---|---|
| 4EBP1 | 1:1000 | Cell Signaling Technology |
| p-4EBP1 | 1:1000 | Cell Signaling Technology |
| S6 | 1:1000 | Cell Signaling Technology |
| p-S6 | 1:1000 | Cell Signaling Technology |
| AMPK | 1:1000 | Cell Signaling Technology |
| p-AMPK | 1:1000 | Cell Signaling Technology |
| Drp1 | 1:1000 | Cell Signaling Technology |
| p-Drp1 | 1:1000 | Cell Signaling Technology |
| ACC | 1:1000 | Cell Signaling Technology |
| p-ACC | 1:1000 | Cell Signaling Technology |
| ACL | 1:1000 | Cell Signaling Technology |
| p-ACL | 1:1000 | Cell Signaling Technology |
| p62 | 1:1000 | Cell Signaling Technology |
| MuRF1 | 1:1000 | Santa Cruz Biotechnology |
| PGC-1α | 1:1000 | GeneTex |
| OPA1 | 1:1000 | Sigma-Aldrich |
| Puromycin | 1:500 | Sigma-Aldrich |
| Anti-Laminin | 5:1000 | Sigma-Aldrich |
| BA-D5 | 1:500 | DSHB, University of Iowa |
| SC-71 | 1:500 | DSHB, University of Iowa |

| **Secondary antibodies** | **Dilution** | **Company** |
|---|---|---|
| Anti-mouse IgG | 1:2500 | Cell Signaling Technology |
| Goat anti-mouse IgG2a | 1:2500 | Cell Signaling Technology |
| Anti-rabbit IgG | 1:2500 | Cell Signaling Technology |
| Rabbit anti-Mouse IgG, Alexa Fluor 488 | 1:1000 | Thermo Fisher Scientific |
| Goat anti-Mouse IgG, Alexa Fluor 555 | 1:1000 | Thermo Fisher Scientific |

**Supplemental Table 3.** *RT-qPCR primers*

| Gene | Gene accession no. | Reference position |
|---|---|---|
| Beta-actin | NM_031144.3 | 1039 |
| IGF-1 | NM_178866.4 | 365 |
| Myomaker | NM_001134517.1 | 1245 |
| MyoD | NM_176079.1 | 1068 |
| Myogenin | NM_017115.2 | 640 |
| Myostatin | NM_019151.1 | 789 |
| MuRF1 | NM_080903.1 | 808 |
| MAFbx | NM_133521.1 | 842 |
| Myf5 | NM_001106783.1 | 163 |
| Pax7 | NM_001191984.1 | 211 |
| Ptch1 | NM_053566.1 | 4213 |
| Gli2 | NM_001107169.1 | 2352 |
| Aplnr | NM_031349.2 | 748 |
| Apln | NM_031612.3 | 551 |
| **Other** | **Forward primer** | **Reverse primer** |
| Piezo 1 | CAC AAA GTA CCG GGC G | AAA GTA GCA CTT GAC G |

## Supplemental Figures

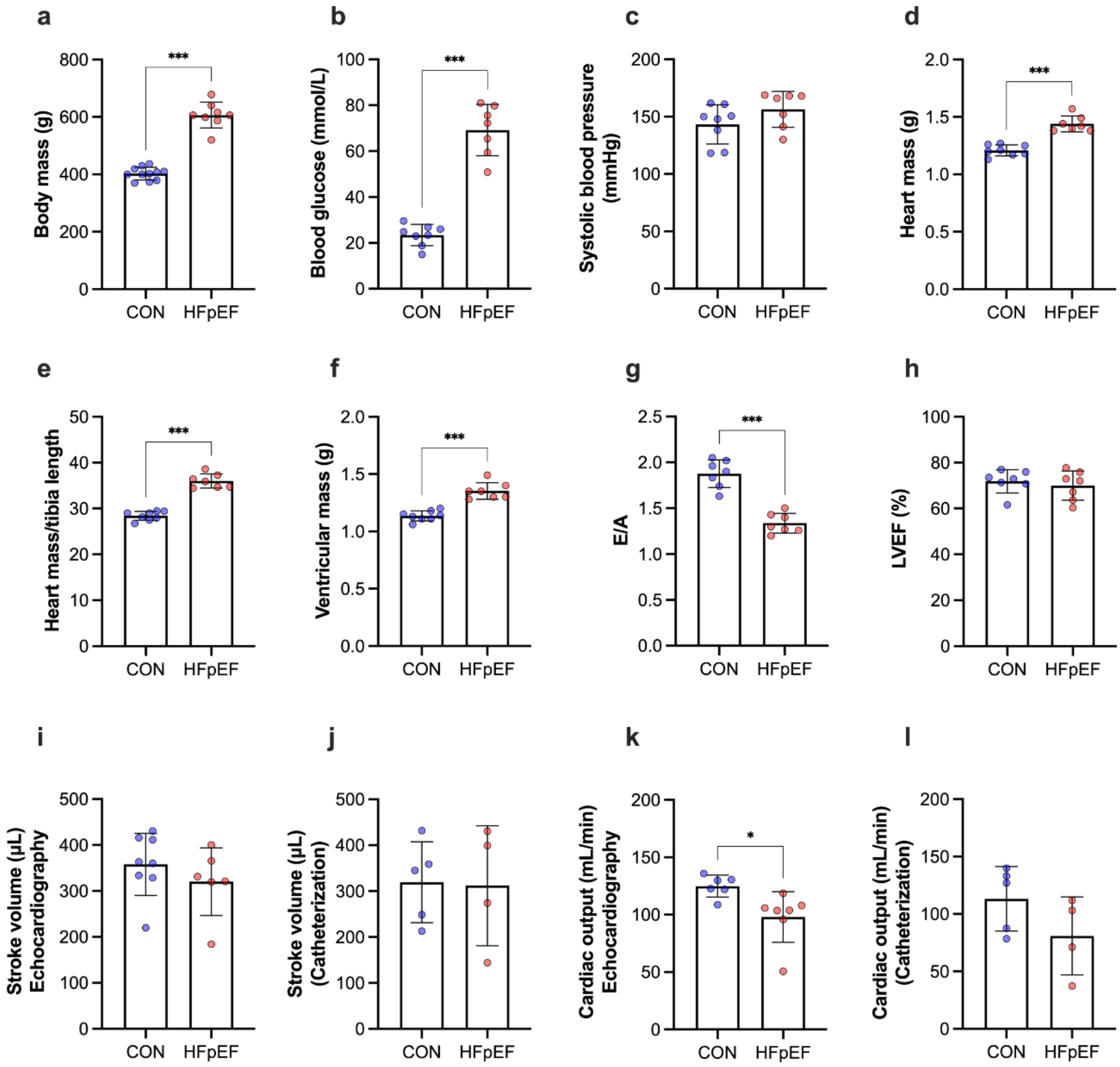


**Supplemental Fig. 1 Cardiometabolic phenotype in HFpEF.** Metabolic features including **a** body mass (CON n = 11, HFpEF n = 8), **b** blood glucose (CON n = 8, HFpEF n = 7) and **c** systolic blood pressure (CON n = 8, HFpEF n = 7). **d** Heart mass (CON n = 8, HFpEF n = 7), **e** heart mass normalized to tibia length (CON n = 8, HFpEF n = 7) and **f** ventricular mass after removing the atria (CON n = 8, HFpEF n = 7). Echocardiographic measurements of **g** early (E wave) and late (A wave) ventricular filling velocities (CON n = 7, HFpEF n = 7), **h** left ventricular ejection fraction (LVEF) (CON n = 7, HFpEF n = 7), **i** stroke volume (CON n = 8, HFpEF n = 6) and **k** cardiac output (CON n = 6, HFpEF n = 7). Invasive hemodynamic measurements of **j** stroke volume (CON n = 5, HFpEF n = 4) and **l** cardiac output (CON n = 5, HFpEF n = 4). Differences between groups were analyzed by unpaired two-tailed Student t-tests. Data are presented as mean±SD, and the level of significance was accepted as * $P < 0.05$, ** $P < 0.01$, *** $P < 0.001$ for all analyses.

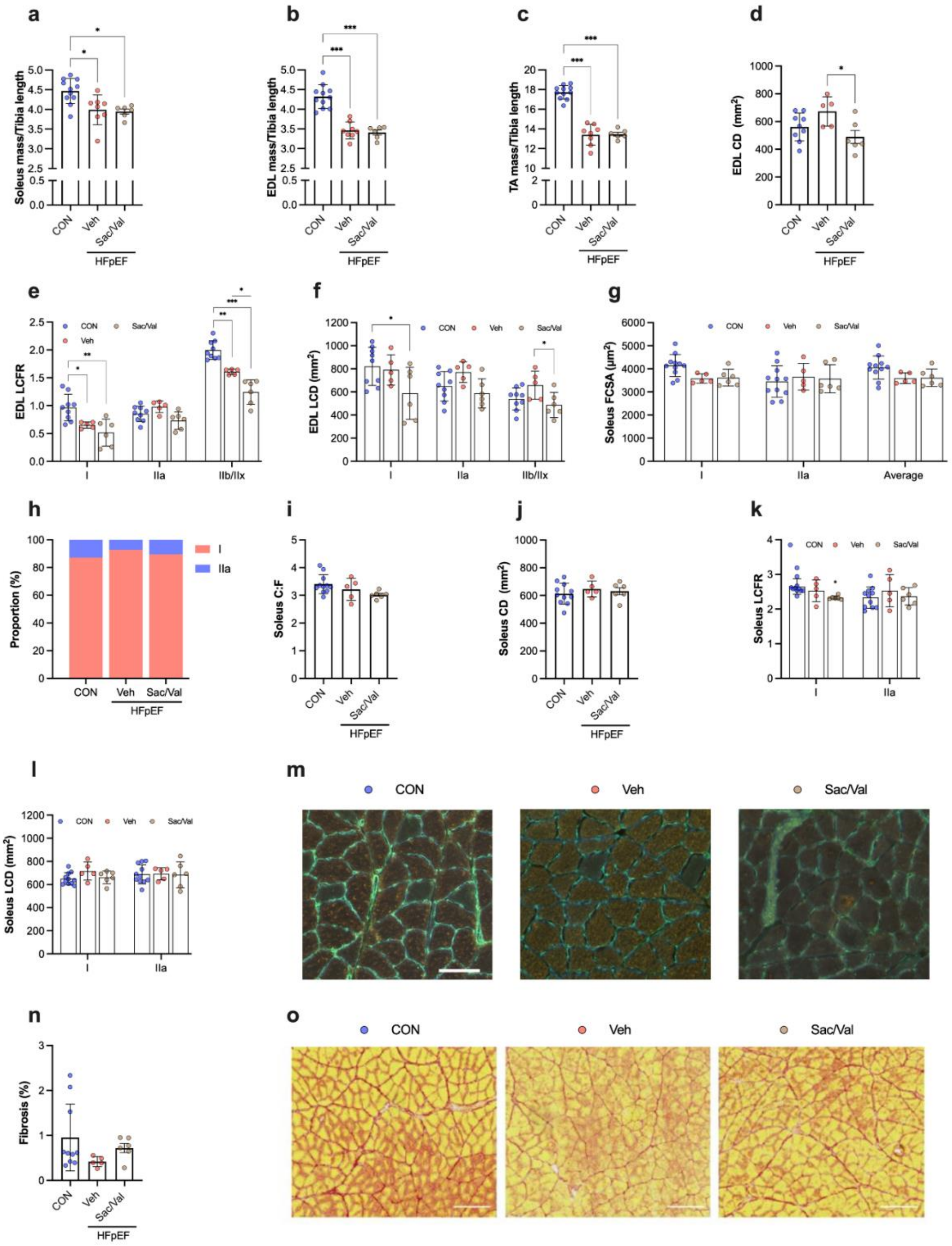


**Supplemental Fig. 2 Skeletal muscle morphology in HFpEF. a** Soleus, **b** EDL and **c** TA mass normalized to tibia length (CON n = 11, HFpEF+Veh n = 8, HFpEF+Sac/Val n = 6). **d** EDL capillary density (CD) (CON n = 9, HFpEF+Veh n = 5, HFpEF+Sac/Val n = 6). EDL **e** local capillary-to-fiber ratio (LCFR) and **f** local capillary density (LCD) (CON n = 9, HFpEF+Veh n = 5, HFpEF+Sac/Val n = 6). Global and fiber-type-specific histological features of the soleus muscle including **g** fiber cross-sectional area (FCSA), **h** fiber type distribution, **i** capillary-to-fiber ratio (C:F), **j** CD, **k** LCFR and **l** LCD (CON n = 11, HFpEF+Veh n = 5, HFpEF+Sac/Val n = 6). **m** Representative images of soleus cryosections stained for Type I (red) and Type IIa (green) fibers and capillaries (green). **n** Percentage of muscle fibrosis (CON n = 10, HFpEF+Veh n = 5, HFpEF+Sac/Val n = 6). **o** Representative images of soleus cryosections stained with Sirius red (fibrotic tissue in red). Between-group differences were assessed by one-way ANOVA followed by Bonferroni *post hoc* test. Data are presented as mean±SD, and the level of significance was accepted as * P < 0.05, ** P < 0.01, *** P < 0.001 for all analyses.

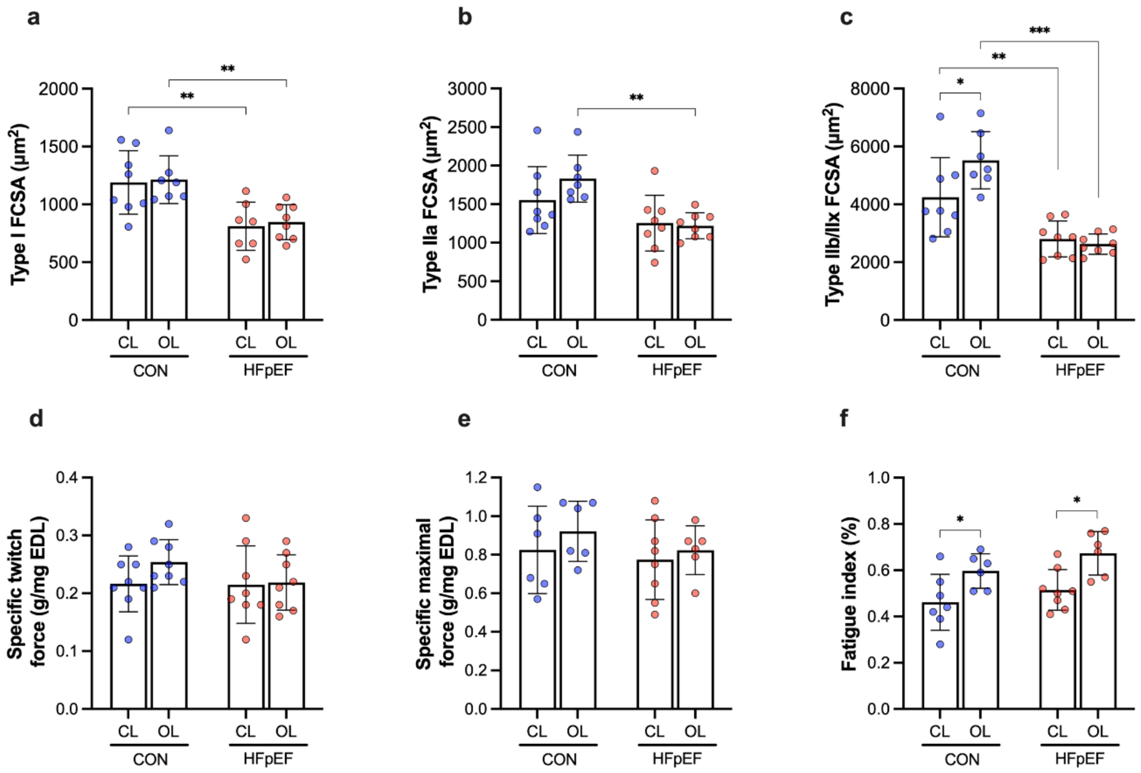


**Supplemental Fig. 3 Overload-induced changes in skeletal muscle morphology and function in HFpEF rats. a** Type I fiber cross-sectional area (FCSA) (CL muscles: CON n = 8 and HFpEF n = 7; OL muscles: CON n = 7 and HFpEF n = 8), **b** Type IIa FCSA (CL muscles: CON n = 8 and HFpEF n = 8; OL muscles: CON n = 7 and HFpEF n = 8) and **c** Type IIb/IIx FCSA (CL muscles: CON n = 8 and HFpEF n = 8; OL muscles: CON n = 7 and HFpEF n = 8) of contralateral (CL) and overloaded (OL) EDL muscles of lean controls (CON) and obese-HFpEF rats. *In situ* functional measurements including for specific force during **d** twitch (CL muscles: CON n = 8 and HFpEF n = 8; OL muscles: CON n = 8 and HFpEF n = 8) and **e** maximal (CL muscles: CON n = 6 and HFpEF n = 8; OL muscles: CON n = 6 and HFpEF n = 6) and **f** fatigability (CL muscles: CON n = 7 and HFpEF n = 8; OL muscles: CON n = 6 and HFpEF n = 6). Differences were assessed by two-way ANOVA followed by Bonferroni *post-hoc* test. Data are presented as mean±SD, and the level of significance was accepted as * P < 0.05, ** P < 0.01, *** P < 0.001 for all analyses.

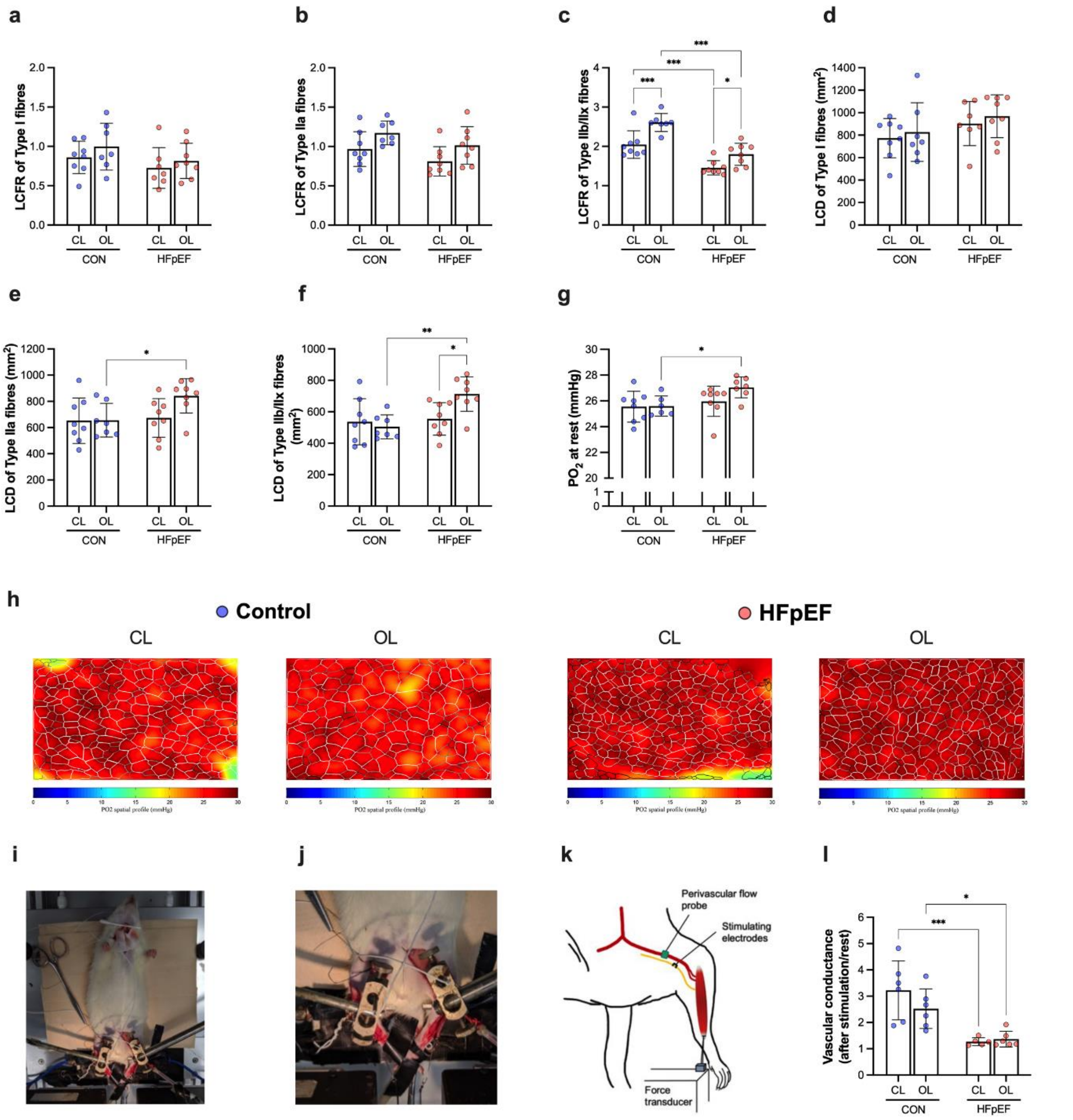


**Supplemental Fig. 4 Blood flow and capillary network morphology.** Local capillary-to-fiber ratio of **a** Type I (CL muscles: CON n = 8 and HFpEF n = 8; OL muscles: CON n = 7 and HFpEF n = 8), **b** Type IIa (CL muscles: CON n = 8 and HFpEF n = 8; OL muscles: CON n = 7 and HFpEF n = 8) and **c** Type IIb/IIx fibers (CL muscles: CON n = 8 and HFpEF n = 8; OL muscles: CON n = 7 and HFpEF n = 8) in contralateral (CL) and overloaded (OL) EDL muscles of controls (CON) and HFpEF rats. Local capillary density (LCD) of **d** Type I (CL muscles: CON n = 8 and HFpEF n = 7; OL muscles: CON n = 7 and HFpEF n = 8), **e** Type IIa (CL muscles: CON n = 8 and HFpEF n = 8; OL muscles: CON n = 7 and HFpEF n = 8) and **f** Type IIb/IIx (CL muscles: CON n = 8 and HFpEF n = 8; OL muscles: CON n = 7 and HFpEF n = 8). **g** Simulation of muscle $PO_2$ at rest (CL muscles: CON n = 8 and HFpEF n = 8; OL muscles: CON n = 6 and HFpEF n = 7) in **h** representative $PO_2$ images. **i-l** Femoral artery set up and vascular conductance during active hyperemia (CL muscles: CON n = 6 and HFpEF n = 5; OL muscles: CON n = 6 and HFpEF n = 6). CL = contralateral muscle, OL = overloaded muscle. Differences were assessed by two-way ANOVA followed by Bonferroni *post-hoc* test. Data are presented as mean±SD, and the level of significance was accepted as * $P < 0.05$, ** $P < 0.01$, *** $P < 0.001$ for all analyses.

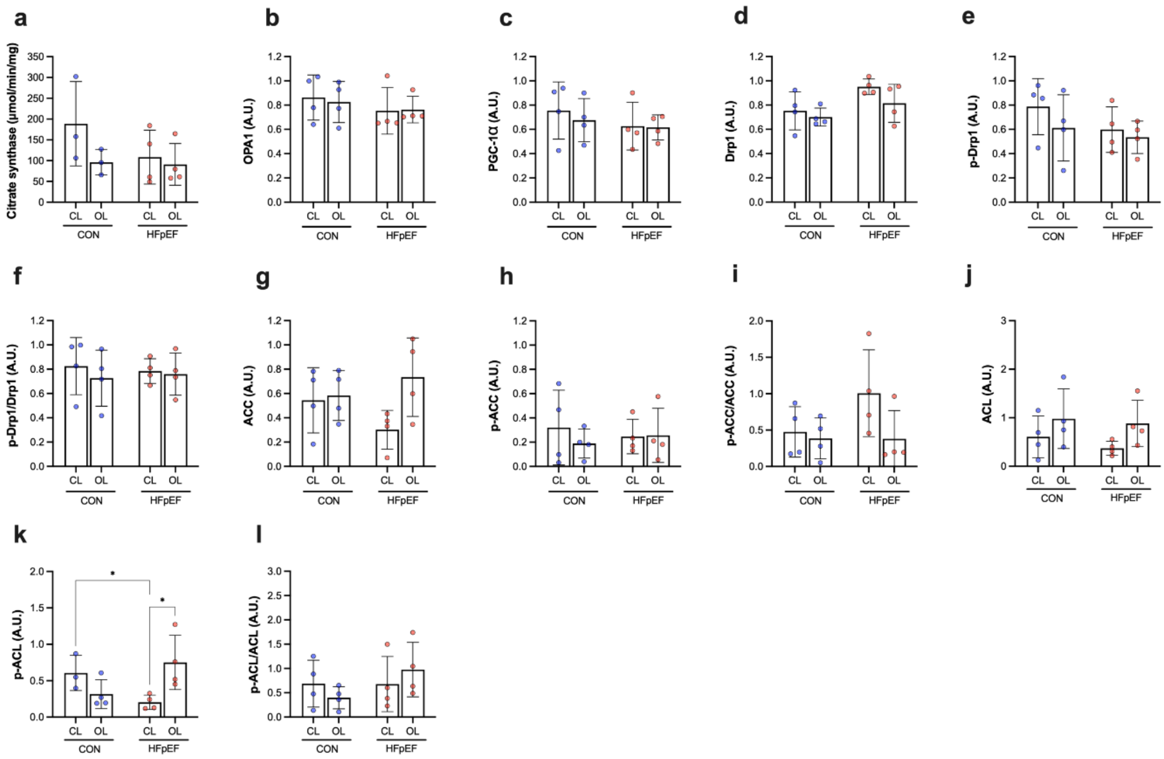


**Supplemental Fig. 5 Overload-induced changes in skeletal muscle mitochondrial proteins. a** Citrate synthase activity (CL and OL muscles: CON n = 3 and HFpEF n = 4). Protein expression (n=4 for CON and HFpEF for OL and CL muscles) of **b** OPA1, **c** PGC-1α, **d** Drp1, **e** phosphorylated (p)-Drp1,**f** pDrp1/Drp1 ratio, **g** ACC, **h** p-ACC, **i** p-ACC/ACC ratio, **j** ACL, **k** p-ACL and **l** p-ACL/ACL. Differences were assessed by two-way ANOVA followed by Bonferroni *post-hoc* test. Data are presented as mean±SD, and the level of significance was accepted as * $P < 0.05$, ** $P < 0.01$, *** $P < 0.001$ for all analyses.

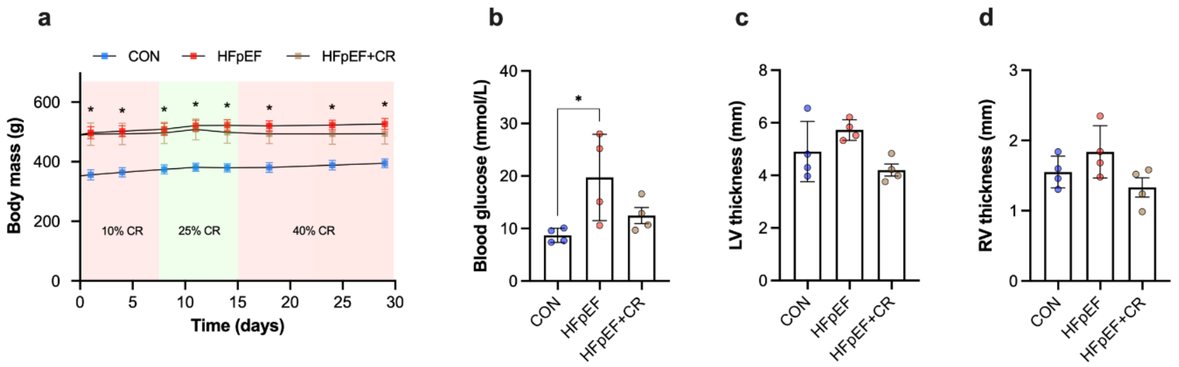


**Supplemental Fig. 6 Cardiometabolic phenotype in HFpEF following caloric restriction.** **a** body mass over time, measured every ~5 days (all n =4). **b** Blood glucose (all n =4). **c** Histological analysis of left ventricular (LV) and **d** right ventricular (RV) thickness (all n =4). Between-group differences were assessed by one-way ANOVA followed by Bonferroni *post-hoc* test. Data are presented as mean±SD, and the level of significance was accepted as * P < 0.05, ** P < 0.01, *** P < 0.001 for all analyses.

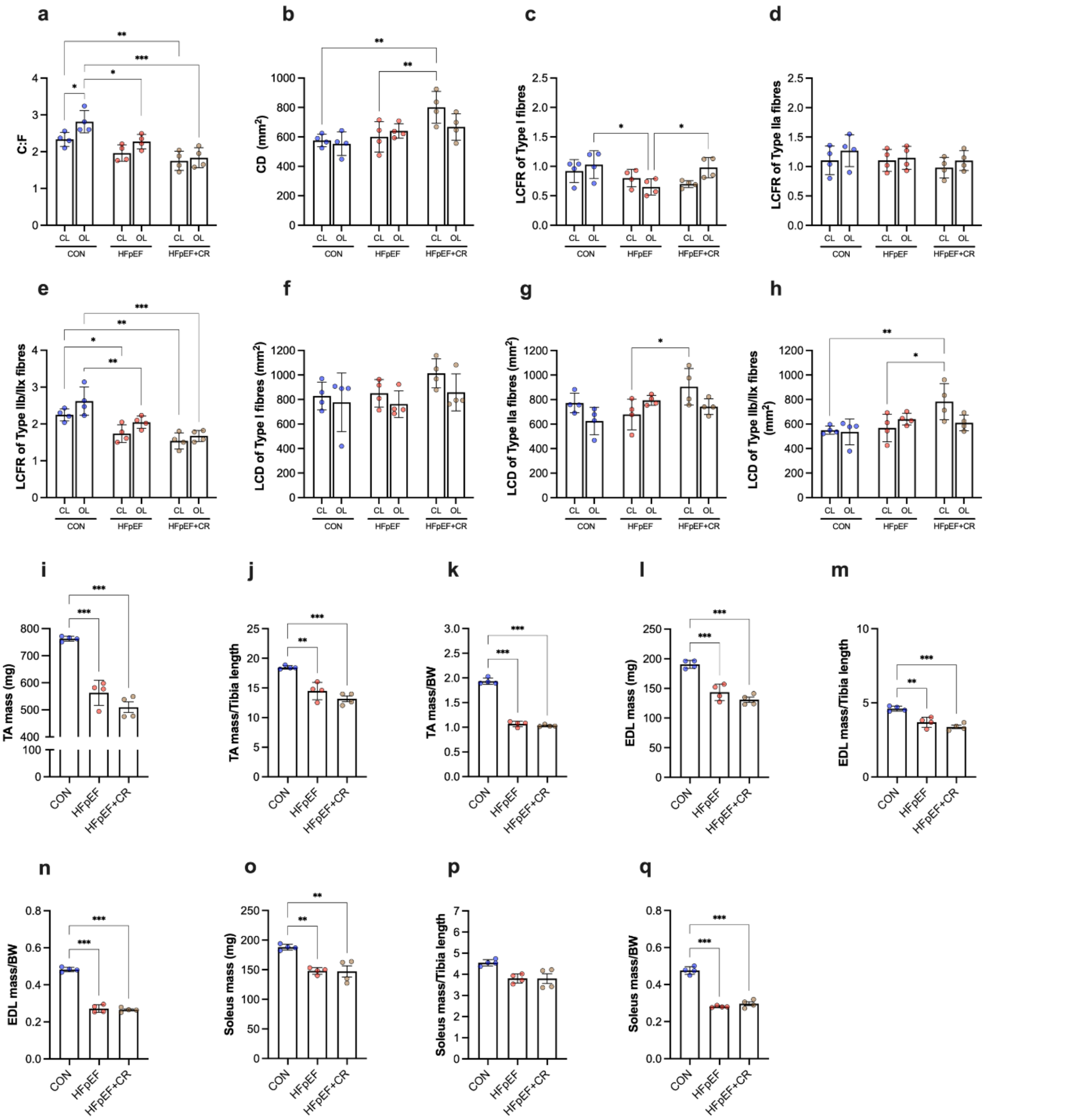


**Supplemental Fig. 7 Effects of caloric restriction on the skeletal muscle remodeling in HFpEF.** Global and fiber-type-specific histological features of the EDL muscle (overloaded (OL) and contralateral (CL) muscles) including **a** capillary-to-fiber (C:F) ratio (C:F), **b** capillary density (CD), local capillary-to-fiber ratio (LCFR) of **c** Type I, **d** Type IIa and **e** Type IIb/IIx fibers and local capillary density (LCD) of **f** Type I, **g** Type IIa and **h** Type IIb/IIx fibers (all n = 4). **i-k** TA, **l-n** EDL and **o-q** soleus mass, represented as total mass and normalized to tibia length and body weight (BW) (all n = 4/group). Differences were assessed by two-way ANOVA followed by Bonferroni *post-hoc* test. Between-group differences were assessed by one-way ANOVA followed by Bonferroni *post hoc* test. Data are presented as mean±SD, and the level of significance was accepted as * P < 0.05, ** P < 0.01, *** P < 0.001 for all analyses.

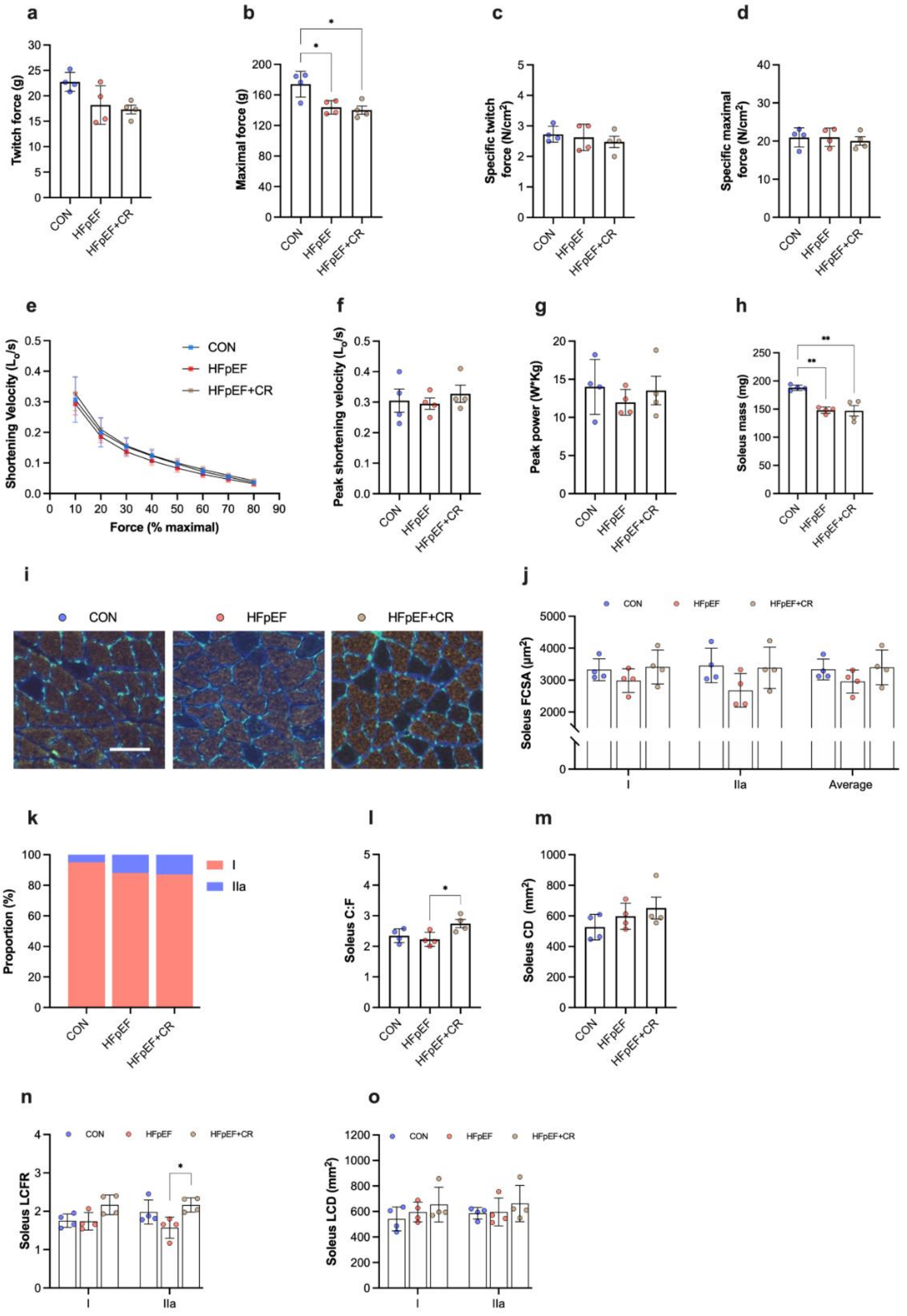


**Supplemental Fig. 8 Effects of caloric restriction on soleus morphology and function in HFpEF.** Absolute **a** twitch and **b** maximal forces. Specific **c** twitch and **d** maximal forces. **e** Force-velocity curve. **f** Peak shortening velocity. **g** Peak power. **h** Muscle mass. **i** Representative images of soleus cryosections stained for Type I (red) and Type IIa (dark green) fibers and capillaries (bright green). **j** Fiber cross-sectional area (FCSA). **k** Fiber type distribution. **l** Capillary-to-fiber ratio (C:F). **m** Capillary density (CD). **n** Local capillary-to-fiber ratio (LCFR). **o** Local capillary density (LCD) (all n = 4). Between-group differences were assessed by one-way ANOVA followed by Bonferroni *post-hoc* test. Data are presented as mean±SD, and the level of significance was accepted as * P < 0.05, ** P < 0.01, *** P < 0.001 for all analyses.

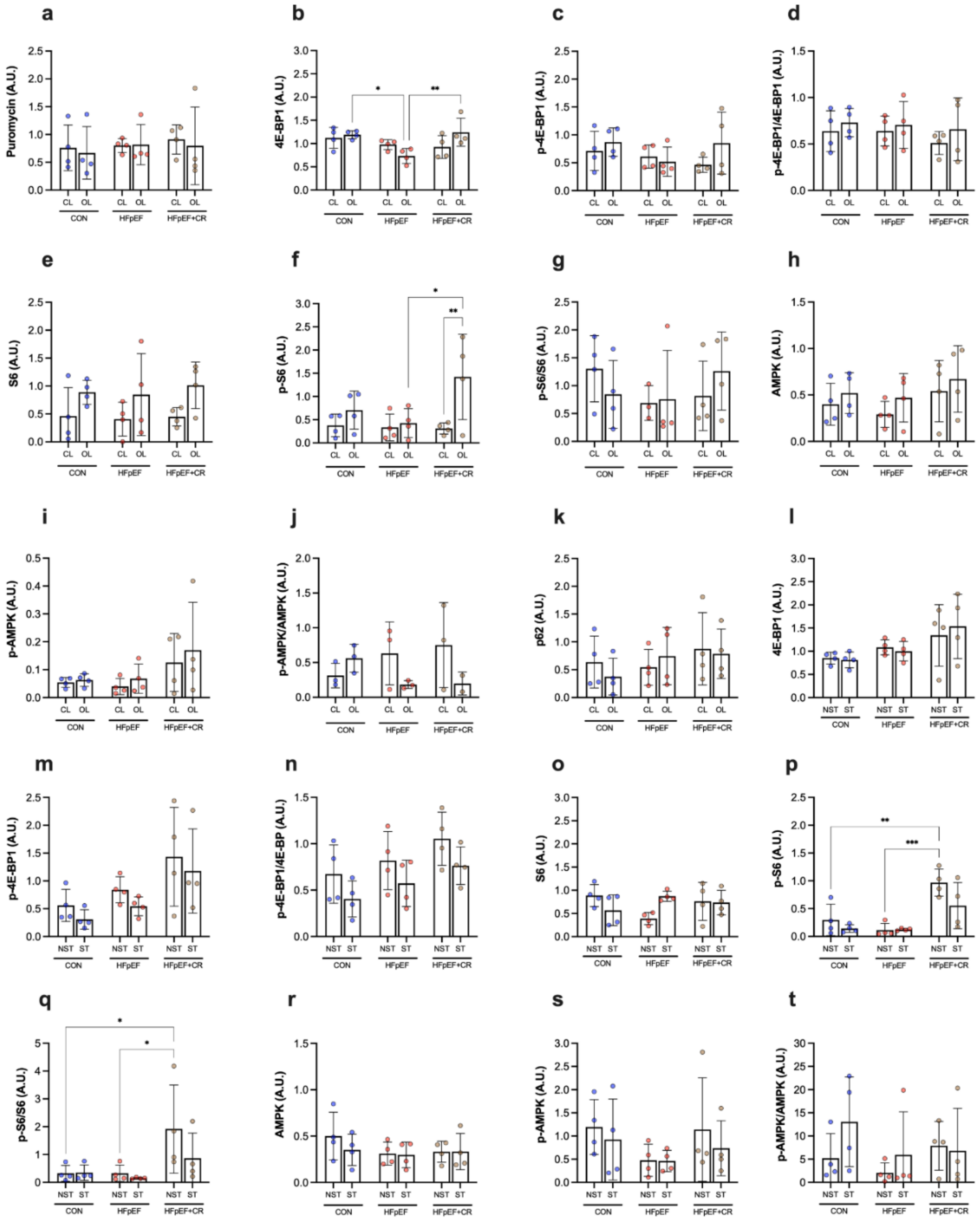


**Supplemental Fig. 9 Anabolic and catabolic signaling pathways.** Protein expression in contralateral (CL) and overloaded (OL) EDL muscles of **a** Puromycin as an index of global protein synthesis, **b** 4E-BP1, **c** phosphorylated (p)-4E-BP1, **d** p-4E-BP1/4E-BP1, **e** S6, **f** p-S6 (all n = 4), **g** p-S6/S6 (CL muscles: CON n = 4, HFpEF n = 3 and HFpEF+CR n = 4; OL muscles: CON n = 4, HFpEF n = 4 and HFpEF+CR n = 4), **h** AMPK, **i** p-AMPK (all n =4), **j** p-AMPK/AMPK (CL muscles: CON n = 3, HFpEF n = 3 and HFpEF+CR n = 3; OL muscles: CON n = 3, HFpEF n = 3 and HFpEF+CR n = 2) and **k** p62 (all n = 4). Protein expression in stimulated (ST) and non-stimulated (NST) soleus muscles of **l** 4E-BP1, **m** p-4E-BP1, **n** p-4E-BP1/4E-BP1, **o** S6, **p** p-S6, **q** p-S6/S6, **r** AMPK, **s** p-AMPK and **t** p-AMPK/AMPK (all n =4). Differences were assessed by two-way ANOVA followed by Bonferroni *post-hoc* test. Data are presented as mean±SD, and the level of significance was accepted as * P < 0.05, ** P < 0.01, *** P < 0.001 for all analyses.

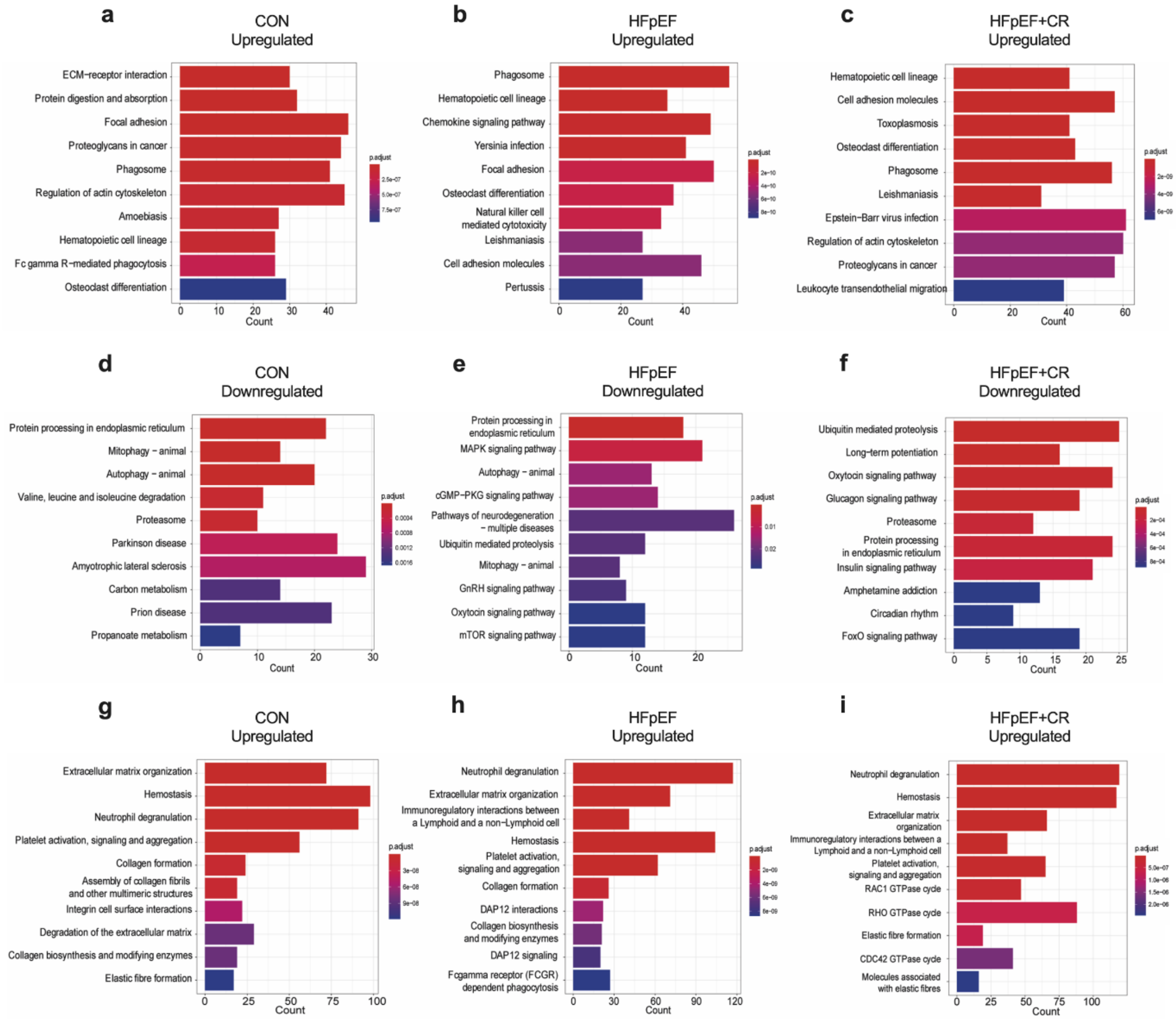


**Supplemental Fig. 10 KEGG and REACTOME pathways analysis between the EDL non-overload *vs.* overload.** KEGG pathway analysis showing **a-c** upregulated and **d-f** downregulated pathways in controls (CON), HFpEF, and HFpEF+CR between the EDL non-overload *vs.* overload (all n = 4). REACTOME analysis of upregulated pathways in **g** CON, **h** HFpEF and **i** HFpEF+CR between contralateral and overloaded muscles (all n = 4). Gene expression levels were quantified by featureCount and normalized by DESeq2 using the negative binomial model. Differentially expressed genes were defined based on adjusted p-value < 0.05. For KEGG pathway analysis, clusterProfiler was used to predict functionally enriched terms and potential pathways involved. The significant terms were selected based on adjusted p-value < 0.05. The adjusted p-value was corrected using Benjamini and Hochberg method.

**Supplemental References**


1. Adams V, *et al.* Ubiquitin-proteasome-system and enzymes of energy metabolism in skeletal muscle of patients with HFpEF and HFrEF. *ESC Heart Fail* **8**, 2556-2568 (2021).

2. Schauer A, *et al.* ZSF1 rat as animal model for HFpEF: Development of reduced diastolic function and skeletal muscle dysfunction. *ESC Heart Fail* **7**, 2123-2134 (2020).

3. Abdellatif M, *et al.* Nicotinamide for the treatment of heart failure with preserved ejection fraction. *Sci Transl Med* **13**, (2021).

4. Bowen TS, *et al.* Exercise Training Reveals Inflexibility of the Diaphragm in an Animal Model of Patients With Obesity-Driven Heart Failure With a Preserved Ejection Fraction. *Journal of the American Heart Association* **6**, (2017).

5. Bowen TS, *et al.* Effects of Endurance Training on Detrimental Structural, Cellular, and Functional Alterations in Skeletal Muscles of Heart Failure With Preserved Ejection Fraction. *J Card Fail* **24**, 603-613 (2018).

6. Franssen C, *et al.* Myocardial Microvascular Inflammatory Endothelial Activation in Heart Failure With Preserved Ejection Fraction. *J Am Coll Cardiol HF* **4**, 312-324 (2016).

7. Goto K, *et al.* Muscular changes in animal models of heart failure with preserved ejection fraction: what comes closest to the patient? *ESC Heart Fail* **8**, 139-150 (2021).

8. Hamdani N, *et al.* Myocardial titin hypophosphorylation importantly contributes to heart failure with preserved ejection fraction in a rat metabolic risk model. *Circ Heart Fail* **6**, 1239-1249 (2013).

9. Satoh T, *et al.* Metabolic Syndrome Mediates ROS-miR-193b-NFYA-Dependent Downregulation of Soluble Guanylate Cyclase and Contributes to Exercise-Induced Pulmonary Hypertension in Heart Failure With Preserved Ejection Fraction. *Circulation* **144**, 615-637 (2021).

10. van Dijk CG, *et al.* Distinct Endothelial Cell Responses in the Heart and Kidney Microvasculature Characterize the Progression of Heart Failure With Preserved Ejection Fraction in the Obese ZSF1 Rat With Cardiorenal Metabolic Syndrome. *Circ Heart Fail* **9**, e002760 (2016).

11. Frischknecht R, Vrbova G. Adaptation of rat extensor digitorum longus to overload and increased activity. *Pflugers Archiv : European journal of physiology* **419**, 319-326 (1991).

12. Thomson DM, Gordon SE. Impaired overload-induced muscle growth is associated with diminished translational signalling in aged rat fast-twitch skeletal muscle. *J Physiol* **574**, 291-305 (2006).

13. Espino-Gonzalez E, *et al.* Abnormal skeletal muscle blood flow, contractile mechanics and fibre morphology in a rat model of obese-HFpEF. *J Physiol* **599**, 981-1001 (2021).

14. Hudlická O, Brown M, Cotter M, Smith M, Vrbová G. The effect of long-term stimulation of fast muscles on their blood flow, metabolism and ability to withstand fatigue. *Pflügers Archiv* **369**, 141-149 (1977).

15. Tickle PG, Hendrickse PW, Degens H, Egginton S. Impaired skeletal muscle performance as a consequence of random functional capillary rarefaction can be restored with overload-dependent angiogenesis. *J Physiol* **598**, 1187-1203 (2020).

16. Bowen TS, *et al.* Heart failure with preserved ejection fraction induces molecular, mitochondrial, histological, and functional alterations in rat respiratory and limb skeletal muscle. *European journal of heart failure* **17**, 263-272 (2015).

17. Garnham JO, *et al.* Chronic heart failure with diabetes mellitus is characterized by a severe skeletal muscle pathology. *J Cachexia Sarcopenia Muscle* **11**, 394-404 (2020).

18. Al-Shammari AA, *et al.* Integrated method for quantitative morphometry and oxygen transport modeling in striated muscle. *J Appl Physiol (1985)* **126**, 544-557 (2019).

19. Liu F, *et al.* Automated fiber-type-specific cross-sectional area assessment and myonuclei counting in skeletal muscle. *J Appl Physiol (1985)* **115**, 1714-1724 (2013).

20. Goodman CA, *et al.* Novel insights into the regulation of skeletal muscle protein synthesis as revealed by a new nonradioactive in vivo technique. *Faseb j* **25**, 1028-1039 (2011).

21. Dobin A, *et al.* STAR: ultrafast universal RNA-seq aligner. *Bioinformatics* **29**, 15-21 (2013).

22. Liao Y, Smyth GK, Shi W. featureCounts: an efficient general purpose program for assigning sequence reads to genomic features. *Bioinformatics* **30**, 923-930 (2013).

23. Love MI, Huber W, Anders S. Moderated estimation of fold change and dispersion for RNA-seq data with DESeq2. *Genome Biol* **15**, 550 (2014).

24. R Development Core Team. A language and environment for statistical computing. Vienna, Austria, Foundation for Statistical Computing (2022)
